# Development and internal validation of time-to-event risk prediction models for major medical complications within 30 days after elective colectomy

**DOI:** 10.1371/journal.pone.0314526

**Published:** 2024-12-02

**Authors:** Janny X. C. Ke, Tim T. H. Jen, Sihaoyu Gao, Long Ngo, Lang Wu, Alana M. Flexman, Stephan K. W. Schwarz, Carl J. Brown, Matthias Görges

**Affiliations:** 1 Department of Anesthesiology, Pharmacology & Therapeutics, Faculty of Medicine, The University of British Columbia, Vancouver, British Columbia, Canada; 2 Department of Anesthesia, St. Paul’s Hospital/Providence Health Care, Vancouver, British Columbia, Canada; 3 Department of Statistics, Faculty of Science, The University of British Columbia, Vancouver, British Columbia, Canada; 4 Department of Biostatistics, Harvard School of Public Health, Boston, Massachusetts, United States of America; 5 Division of General Medicine, Beth Israel Deaconess Medical Center, Harvard Medical School, Boston, Massachusetts, United States of America; 6 Department of Surgery, Faculty of Medicine, The University of British Columbia, Vancouver, British Columbia, Canada; 7 Department of Surgery, St. Paul’s Hospital/Providence Health Care, Vancouver, British Columbia, Canada; 8 BC Children’s Hospital Research Institute, Vancouver, British Columbia, Canada; Osaka International Cancer Institute: Osaka Kokusai Gan Center, JAPAN

## Abstract

**Background:**

Patients undergoing colectomy are at risk of numerous major complications. However, existing binary risk stratification models do not predict when a patient may be at highest risks of each complication. Accurate prediction of the timing of complications facilitates targeted, resource-efficient monitoring. We sought to develop and internally validate Cox proportional hazards models to predict time-to-complication of major complications within 30 days after elective colectomy.

**Methods:**

We studied a retrospective cohort from the multicentered American College of Surgeons National Surgical Quality Improvement Program procedure-targeted colectomy dataset. Patients aged 18 years or above, who underwent elective colectomy between January 1, 2014 and December 31, 2019 were included. *A priori* candidate predictors were selected based on variable availability, literature review, and multidisciplinary team consensus. Outcomes were mortality, hospital readmission, myocardial infarction, cerebral vascular events, pneumonia, venous thromboembolism, acute renal failure, and sepsis or septic shock within 30 days after surgery.

**Results:**

The cohort consisted of 132145 patients (mean ± SD age, 61 ± 15 years; 52% females). Complication rates ranged between 0.3% (n = 383) for cardiac arrest and acute renal failure to 5.3% (n = 6986) for bleeding requiring transfusion, with readmission rate of 8.6% (n = 11415). We observed distinct temporal patterns for each complication: the median [quartiles] postoperative day of complication diagnosis ranged from 1 [0, 2] days for bleeding requiring transfusion to 12 [6, 18] days for venous thromboembolism. Models for mortality, myocardial infarction, pneumonia, and renal failure showed good discrimination with a concordance > 0.8, while models for readmission, venous thromboembolism, and sepsis performed poorly with a concordance of 0.6 to 0.7. Models exhibited good calibration but ranges were limited to low probability areas.

**Conclusions:**

We developed and internally validated time-to-event prediction models for complications after elective colectomy. Once further validated, the models can facilitate tailored monitoring of high risk patients during high risk periods.

**Trial registration:**

Clinicaltrials.gov (NCT05150548; Principal Investigator: Janny Xue Chen Ke, M.D., M.Sc., F.R.C.P.C.; initial posting: November 25, 2021)

## Introduction

In North America, approximately 300 000 planned or emergent colectomies (large bowel resections) are performed yearly [1, 2], with over 50% performed in patients aged 65 and older [3]. Indications for elective colectomies include colorectal cancer (41%), diverticulitis (23%), inflammatory bowel disease (8%), and non-malignant colorectal polyps (12%) [4]. The median length of hospital stay is 6 days [5, 6], with a mean cost of stay of $25 900 United States dollars [1]. Postoperative mortality and morbidity rates within 30 days of operation are 1.4% and 12.4%, respectively [7]. Common postoperative surgical complications include ileus (12%), bleeding (8%), surgical site infection (7%), and anastomotic leakage (5%) [4], while major medical complications include pneumonia (2%), venous thromboembolism (1 to 2%), myocardial infarction (< 1%), acute renal failure (0.4%), and cerebrovascular accident (0.2%) [8]. Postoperative complications are associated with increased lengths of stay, readmissions, and costs, and prognosticate both short- and long-term mortality [9–12].

As the population ages and becomes more frail with increased risk of perioperative morbidity [13], prediction models that identify high-risk patients allow both preoperative shared decision-making and postoperative targeted enhanced monitoring of complications. Accurate risk prediction may facilitate timely treatment and reduce failure to rescue [14]. A systematic review found 26 models developed specifically for patients aged 65 years or older undergoing colorectal cancer surgery [15]. Other prediction models include the American College of Surgeons National Surgical Quality Improvement Program (NSQIP) surgical risk calculator derived from the multicentered North American dataset [16] and the Codman score derived from a single-centre South African dataset [17]. However, existing models are limited by moderate to high risks of bias [15], incomplete reporting of calibration [15], or poor performance during external validation [18, 19]. Furthermore, existing risk prediction models consider outcome variables as dichotomous (i.e., occurred *vs* did not occur within a specified period) without predicting *when* such complications may occur. It may be overwhelming in clinical practice for clinicians, patients/caregivers, and health systems to monitor for all possible complications throughout the postoperative period. Accurate prediction of the timing of when patients are at the highest risks for different complication allows for tailored monitoring strategies and efficient resource use [12, 20], particularly during the post-discharge period.

Our study objective was to develop and internally validate distinct time-to-event risk prediction models for each of the following outcomes within 30 days after elective colectomy: mortality, hospital readmission, myocardial infarction, cerebral vascular event, pneumonia, venous thromboembolism, acute renal failure, and sepsis or septic shock.

## Materials and methods

Following approval from the Providence Health Care Research Ethics Board with waiver of informed consent (H21-02670; November 16, 2021), we conducted survival analysis modelling using a retrospective linked cohort from the prospectively collected multicentered NSQIP general and Procedure Targeted Colectomy datasets [21]. Before data analysis, the study protocol was registered at clinicaltrials.gov (NCT05150548; November 25, 2021). Data were accessed for analysis on January 25, 2022, and are reported according to the transparent reporting of a multivariable prediction model for individual prognosis or diagnosis (TRIPOD) guidelines [22].

### Study population

The study included patients aged 18 years or above who underwent elective colectomy between January 1, 2014 and December 31, 2019, in the NSQIP Procedure Targeted Colectomy dataset (with linkage to the NSQIP general dataset). We excluded patients who underwent urgent or emergent surgeries and surgeries with a non-elective nature (acute diverticulitis, enterocolitis, or volvulus). We also excluded patients with disseminated cancer, pre-existing wound infection, systemic sepsis, preoperative ventilator dependence, or American Society of Anesthesiologists (ASA) Physical Status V.

### Model endpoints

We created a distinct time-to-event prediction model for each of the following outcomes within 30 days postoperatively: mortality, hospital readmission, myocardial infarction, cerebral vascular event, pneumonia, venous thromboembolism, acute renal failure, and sepsis or septic shock. NSQIP definitions of the variables used are listed in S1 Appendix.

### Candidate predictors

We selected *a priori* candidate predictors based on variable availability (both in the NSQIP database and routinely collected in hospital electronic health records for future external validation and generalizability), literature review for clinical relevance, and multidisciplinary team consensus. Candidate predictors were age, sex, race, body mass index, American Society of Anesthesiologists (ASA) Physical Status, diabetes, smoker within one year preoperatively, severe chronic obstructive pulmonary disease, ascites, congestive heart failure, hypertension on medications, preoperative renal failure, preoperative functional status, dyspnea, coagulopathy, preoperative steroid or immunosuppressant use, chemotherapy within 90 days preoperatively, surgical approach, total operation time, use of any regional anesthesia, wound classification at the end of surgery, and the primary indication for colectomy.

### Sample size calculation

We used the pmsampsize package (version 1.1.3) in R (R Foundation for Statistical Computing, Vienna, Austria) and followed guidance for time-to-event multivariable modelling [23]. Based on an event rate of 0.01 over 30 days for the rarest outcomes: myocardial infarction and mortality (i.e., 0.00033 per patient per day in the study period totalling 30 days), a Cox-Snell R-squared 0.11 (i.e. the maximum possible for an event rate of 0.01 over 30 days), an acceptable difference of 0.05 in apparent & adjusted R-squared, a shrinkage of 0.9, we required a minimum of 51 events (5091 patients, 152 730 person-days of follow-up) for modelling with 30 predictor variables [23].

### Statistical analysis

Data were analyzed using R, with open-source codes provided on Open Science Framework (https://osf.io/6y8fs/?view_only=855a559f448e48e99827c6e20da8e1c6). We converted age from the NSQIP character variable to a continuous numeric variable, with ages over 90 years (“90+”) recorded as 90. We standardized the NSQIP notation for missing values (“-99”, “unknown”, “None Assigned”, or “NULL”). For the pre-processing of outcome variables for survival analysis, we created a censored variable for each outcome to be modelled based on time-to-event and whether the endpoint occurred. For cohort characteristics, continuous variables are presented as mean ± SD and median [quartiles] for parametric and nonparametric data, and categorical variables are represented as frequency and percentage. Distributions of time-to-complication are reported using cumulative hazard plots. We explored co-occurrence of model endpoints using a heat map.

#### Missing data

For candidate predictors, missing values were quantified and treated as follows by the percentage of missingness: if more than 10% were missing, the predictor would be excluded from the model; if ≤ 1% or fewer were missing, complete case analysis would be performed; if between greater than 1% and ≤ 10% were missing, multiple imputation by chained equations would be performed. For outcome variables, any missing values were coded as negative for the complication. Patients who suffered a complication but did not have time-to-event data were excluded from modelling for the given complication.

#### Model development

We constructed a multivariable Cox proportional hazards model for each outcome using *a priori* candidate predictors listed above. We assessed potential multicollinearity through variance inflation factor, with > 4 indicating significant collinearity. The collinear variables were aggregated if feasible; otherwise, the variable with fewer missing values or that which could be more accurately ascertained was kept. No predictor selection was performed during modelling to avoid bias and overfitting [24, 25].

Assumptions for the Cox proportional hazards models were checked. Hazard proportionality was checked via visual inspection of the Kaplan-Meier curves, Log-Log S(t) test, and Schoenfeld residuals. Linearity between log hazard and continuous predictors was checked using residual plots. In Log-Log S(t) tests and Cox proportional hazards models, time is shifted by one day to avoid computational issues when taking the logarithm.

For sensitivity analysis, we compared the performances of Weibull accelerated failure time models to Cox proportional hazards models, as the former directly models failure time and may be more interpretable and robust in cases of omitted predictor variables or if assumptions for Cox proportional hazards models were not met [26].

#### Model validation

We assessed the internal validity of the prediction models using bootstrap validation with 500 repetitions. We used the following performance measures as per recommendations [27, 28]:

1. Harrell’s concordance index and Uno’s concordance index: Briefly, concordance index in survival analysis is derived from paired comparisons of subjects within the sample, and it represents the extent to which a model correctly assigns higher risk to a subject who had an event earlier compared to another subject who had an event later (or who did not experience the event at all). A concordance index of 1 represents a perfect match between risks and event times, while a concordance index of 0.5 represents random assignments [29]. Uno’s concordance index further accounts for right-censoring based on inverse probability of censoring weights [30].
2. Time-dependent area under the receiver operating characteristic curve (time-dependent AUC): Time-dependent AUC evaluates how well a survival model can distinguish between those who will or will not experience an event at a specified time point [27].
3. Time-dependent Brier score: The time-dependent Brier score in survival analysis quantifies the mean squared difference between predicted probabilities and observed outcomes, thus reflecting both discrimination and calibration [27]. It is adjusted for right censoring using inverse probability of censoring weighting [31]. As the Brier scores depend on the incidence of the outcome, they are scaled by the maximum score for a non-informative model to 1 being no discrepancy between predicted and observed probabilities, and 0 being the model with no improvement from a non-informative model [32].
4. Cox and Snell’s pseudo R-squared: The pseudo R-squared quantifies the proportional reduction in uncertainty when moving from the baseline model (of intercept alone) to the full model [23].
5. Calibration plots: Calibration plots offer insights into the consistency and accuracy of model predictions by comparing predicted probabilities against observed outcomes. These plots were generated using bootstrapped samples with 200 repetitions, binned into deciles, and included 95% confidence intervals.

## Results

### Cohort characteristics

**Fig 1:**
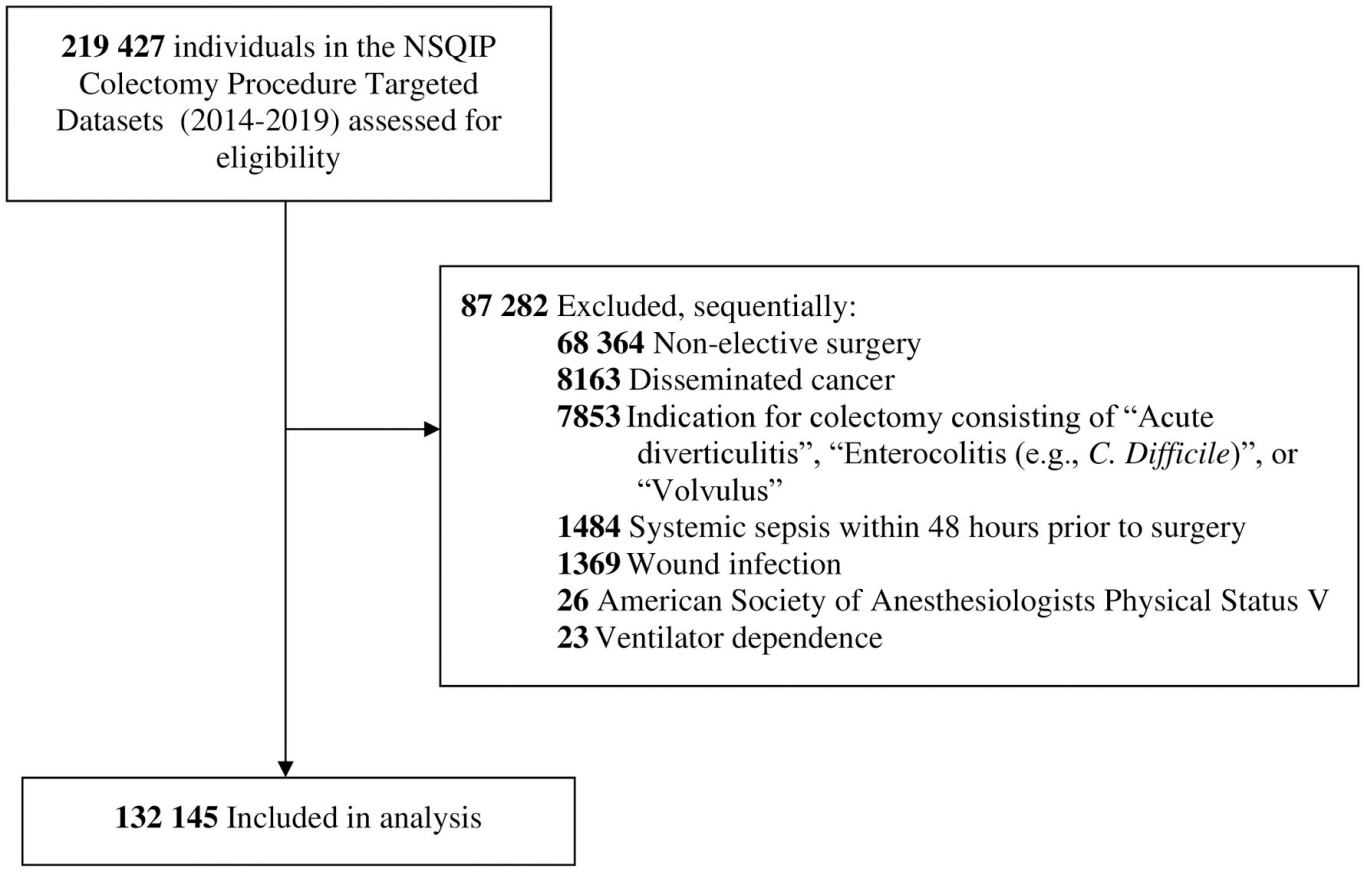
Study cohort flow diagram.

**Fig 2:**
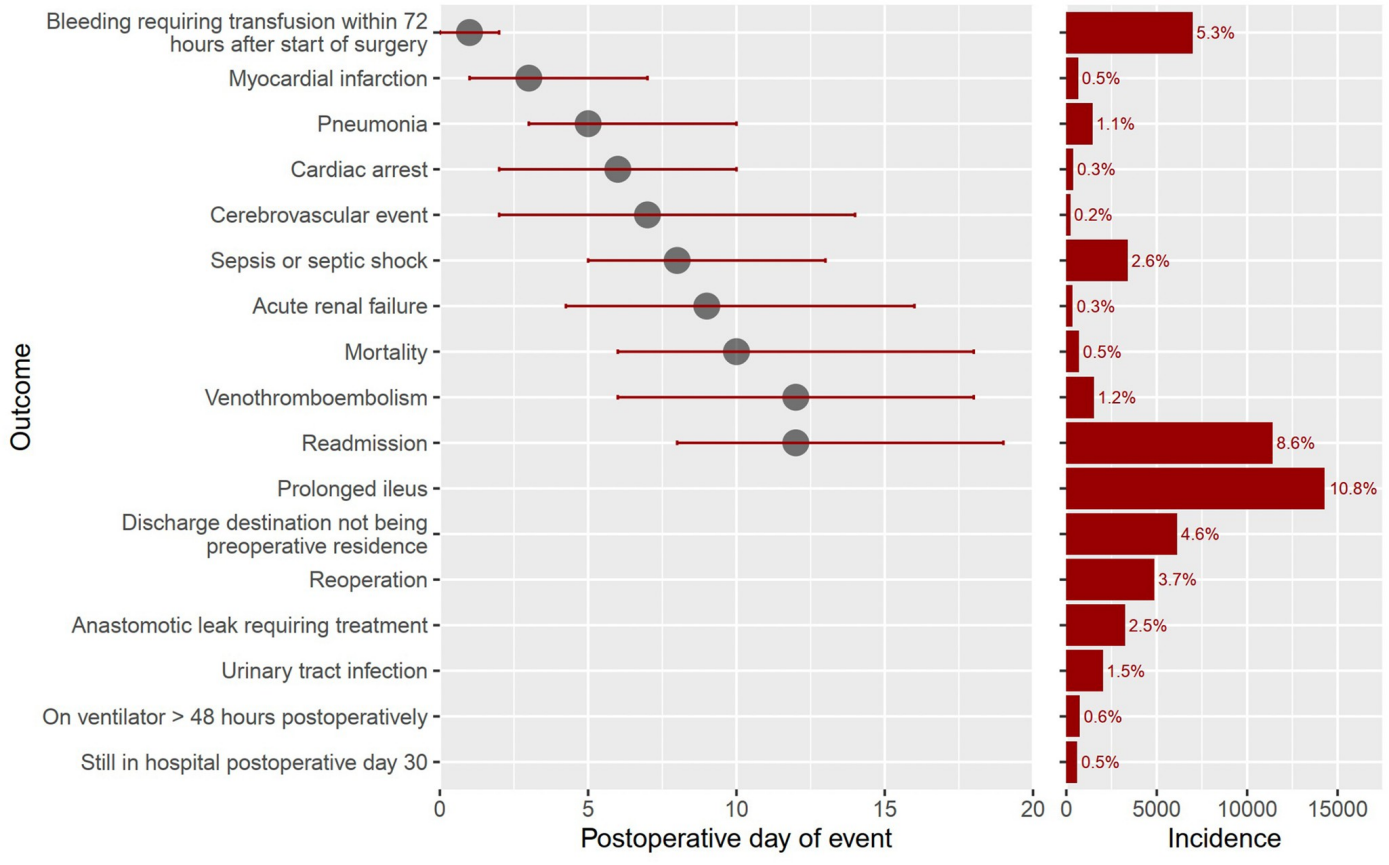
Incidence and timeline of postoperative morbidity and mortality. The incidence and median [quartiles] of postoperative day of events are presented for each postoperative complication. See S2.1 Table in S2 Appendix.

**Fig 3:**
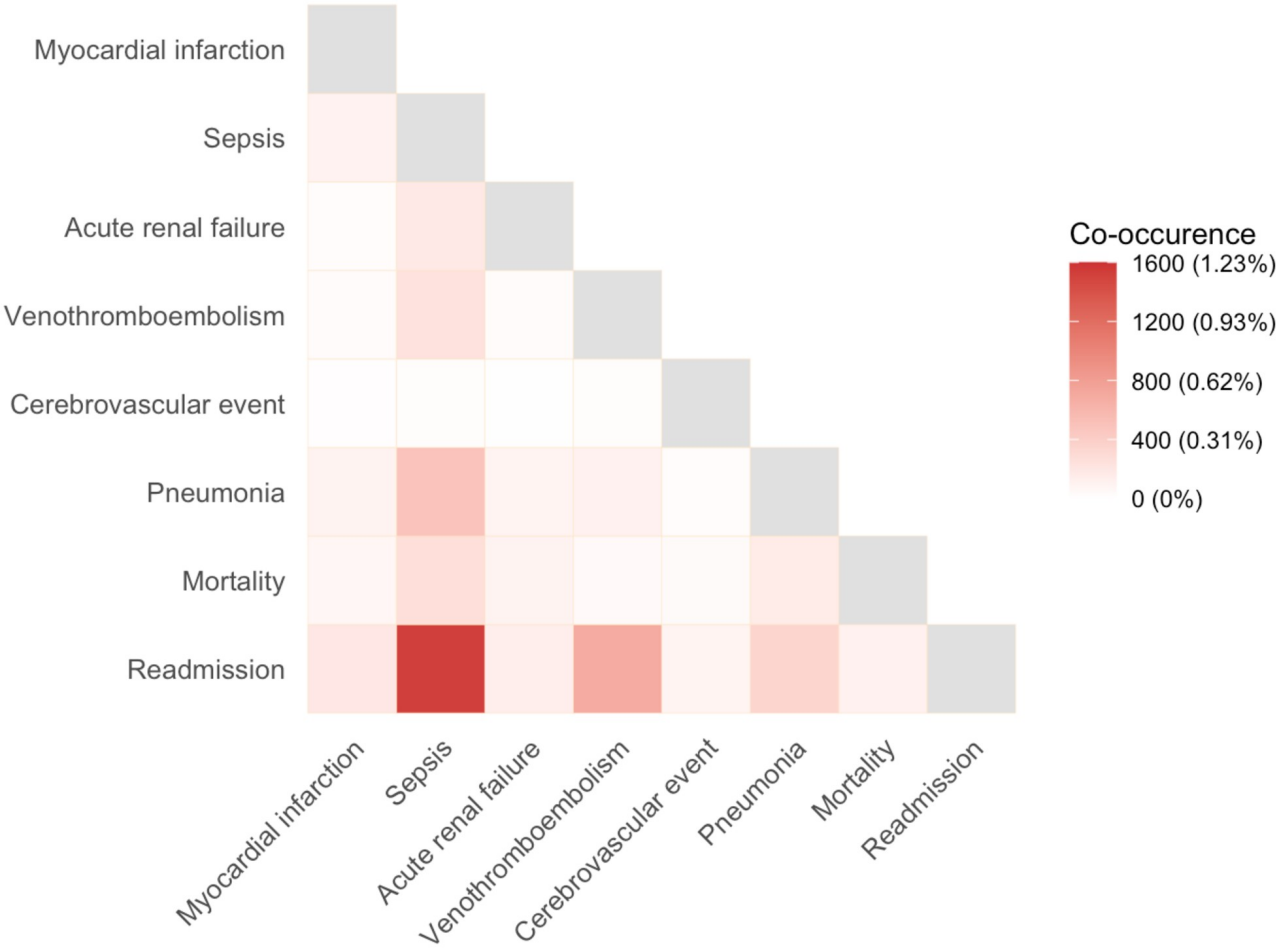
Dendrogram of co-occurrence of model endpoints. High co-occurrence is represented by dark red while low co-occurrence is represented by a light red and no co-occurrence by white.

After applying exclusion criteria, the final cohort contained 132 145 patients (Fig 1). Cohort characteristics are shown in Table 1; 47.8% were male (63 199/132 145), with a mean ± SD age of 61 ± 14 years. The median [quartiles] length of stay was 4 [3, 6] days. Incidence and timing of postoperative morbidity and mortality are reported in Fig 2 and S2.1 Table in S2 Appendix. The incidence of death within 30 days was 0.5% (714/132 145), with the median [quartiles] time to death at 10 [6, 18] days postoperatively. The incidences of readmission, bleeding requiring transfusion within 72 hours, and sepsis or septic shock were 8.6%, 5.6%, and 2.6%, respectively, while other model endpoints had incidences less than 2%. Cumulative hazard plots for each model endpoint are shown in S1 Fig in S3 Appendix. Heat map indicated highest co-occurrences between sepsis and readmission, venous thromboembolism and readmission, and sepsis and pneumonia (Fig 3).

**Table 1:** Cohort characteristics.

Variable	*N = 132*,*145*	*Missing N (%)*
***Preoperative***		
Age, mean (SD), y	61 (14)	0
Male sex, No. (%)	63 199 (47.8)	0
ASA PS, No. (%)		129 (0.1)
I	2733 (2.1)	
II	60 883 (46.1)	
III	63 911 (48.4)	
IV	4489 (3.4)	
Race, No. (%)		18367 (13.9)
American Indian or Alaska Native	540 (0.4)	
Asian	4188 (3.2)	
Black or African American	11 275 (8.5)	
Native Hawaiian or Pacific Islander	295 (0.2)	
White	97 480 (73.8)	
Primary indication for colectomy, No. (%)		114 (0.1)
Bleeding	219 (0.2)	
Cancer	62 260 (47.1)	
Diverticulitis	25754 (19.5)	
Inflammatory bowel disease	10 720 (8.1)	
Non-malignant polyp	15835 (12.0)	
Other	17 243 (13.0)	
Steroid/immunosuppressant use for a chronic condition, No. (%)	12 142 (9.2)	1233 (0.9)
Body mass index, median (IQR), kg·m^−2^	28 (24–32)	428 (0.3) for Height 271 (0.2) for Weight
Ascites within 30 days preoperatively, No. (%)	185 (0.1)	0
Chemotherapy within 90 days preoperatively, No. (%)	7224 (5.5)	1335 (1.0)
Dyspnea, No. (%)		0
At rest	286 (0.2)	
With moderate exertion	7510 (5.7)	
Preoperative functional health status, No. (%)		430 (0.3)
Independent	130 195 (98.5)	
Partially dependent	1350 (1.0)	
Totally dependent	170 (0.1)	
Smoker within one year preoperatively, No. (%)	20 230 (15.3)	0
History of severe COPD, No. (%)	5507 (4.2)	0
Bleeding disorders or on anticoagulants, No. (%)	2533 (1.9)	0
Congestive heart failure, within 30 days before surgery, No. (%)	728 (0.6)	0
Hypertension requiring medication, No. (%)	62 266 (47.1)	0
Preoperative renal failure (preoperative acute renal failure, dialysis, and/or GFR ≤ 60 mL·min^−2^), No. (%)	18 103 (13.7)	0 for acute renal failure 0 for dialysis 11503 (9) for GFR
Diabetes mellitus, No. (%)		0
On insulin	6154 (4.7)	
On non-insulin medication	14 304 (10.8)	
***Intraoperative***		
Any regional anesthesia use, No. (%)	14 732 (11.1)	0
Total operating time, median (IQR), min	171 [123–234]	9 (0.0)
Operative approach, No. (%)		37 (0.0)
Minimally invasive	56 032 (42.4)	
Minimally invasive with open- or hand-assist	42 732 (32.3)	
Minimally invasive with unplanned conversion to open	9373 (7.1)	
Open	23 897 (18.1)	
Other	74 (0.1)	
Wound classification, No. (%)		0
Clean	1311 (1.0)	
Clean/contaminated	109 950 (83.2)	
Contaminated	14686 (11.1)	
Dirty/infected	6198 (4.7)	
***Postoperative***		
Length of stay, median (IQR), d	4 (3–6)	295 (0.2)
Discharge destination, No. (%)		251 (0.2)
Against medical advice	54 (0.0)	
Expired	480 (0.4)	
Facility which was home	556 (0.4)	
Home	125 149 (94.7)	
Hospice	36 (0.0)	
Multi-level senior community	12 (0.0)	
Rehabilitation	1640 (1.2)	
Separate acute care	259 (0.2)	
Skilled care, not home	3616 (2.7)	
Unskilled facility not home	92 (0.1)	
Anastomotic leak, No. (%)		298 (0.2)
Leak, no treatment intervention documented	263 (0.2)	
Leak, treated w/ interventional means	879 (0.7)	
Leak, treated w/ non-interventional/non-operative means	535 (0.4)	
Leak, treated w/ reoperation	1831 (1.4)	

Detailed definitions are given in S1 Appendix. ASA PS, American Society of Anesthesiologists Physical Status; COPD, chronic obstructive pulmonary disease; GFR, glomerular filtration rate as calculated using Chronic Kidney Disease Epidemiology Collaboration (CKD-EPI) formula; IQR, interquartile range.

### Model development

Race was removed as a predictor as 13.9% of patients had unknown or unreported information (Table 1). The remaining predictors had ≤ 1% missing values (Table 1); thus, complete case analysis was performed without requiring any imputation. We did not find concerns for multicollinearity among predictors, with the maximum variance inflation factor being 2.3. Some predictors did not meet the proportional hazards or linearity assumptions for certain models (S2.2 Table in S2 Appendix), particularly for sepsis and readmission, which may indicate further complexities in the underlying relationships amongst predictors and outcomes. We did not remove these predictors as that would be a form of variable selection that may add bias [24, 25]. Nevertheless, we addressed this limitation using bootstrap [33], which showed that the estimated hazard ratios and standard errors remain consistent across bootstrapped samples. Also, the results of Cox proportional hazards models were similar to accelerated failure time models, which are not as affected by predictors not meeting the proportional hazard assumptions [26]. For model stability, we removed rare categories of categorical variables where no events were observed (S2.4, S2.6, and S2.8 Tables in S2 Appendix).

### Model results

The formula, baseline hazard function, and coefficients for each model are reported in S2.3-S2.11 Tables in S2 Appendix. The performance measures for the Cox proportional hazards models are listed in Table 2 with calibration plots shown in S3 Appendix.

**Table 2:** Performance measures of Cox proportional-hazard models.

Model	*N*	Harrell’s concordance index (SE)^a^	Uno’s concordance index	Time-dependent AUROC at 30 days (SE)^a^	Cox and Snell’s Pseudo R-squared	Maximum Possible Pseudo R-squared ^b^	Time-dependent Brier score at 30 days	Scaled time-dependent Brier score at 30 days
**Mortality**	129 606	0.846 (0.0064)	0.845	0.835 (0.0062)	0.0099	0.1176	0.0052	0.0209
**Myocardial infarction**	129 677	0.813 (0.0069)	0.813	0.802 (0.0076)	0.0067	0.1084	0.0048	0.0088
**Acute renal failure**	129 606	0.806 (0.0111)	0.805	0.800 (0.0102)	0.0038	0.0609	0.0027	0.0053
**Cerebrovascular event**	126 943	0.802 (0.0131)	0.802	0.796 (0.0125)	0.0023	0.0392	0.0017	0.0041
**Pneumonia**	129 677	0.773 (0.0060)	0.772	0.762 (0.0056)	0.0115	0.2252	0.0105	0.0170
**Sepsis**	129 676	0.679 (0.0046)	0.679	0.670 (0.0044)	0.0111	0.4532	0.0247	0.0142
**Venous Thromboembolism**	129 676	0.655 (0.0070)	0.655	0.648 (0.0065)	0.0036	0.2378	0.0114	0.0042
**Readmission**	129 643	0.617 (0.0028)	0.617	0.789 (0.0077)	0.0149	0.8680	0.0776	0.0165

AUROC, area under the receiver operating characteristic curve; *N*, total number of patients; *SE*, standard error

^a^ Calculated using bootstrapping with 500 repetitions.

^b^ Maximum possible Pseudo R-squared is calculated by 1 –exp(2 * loglik_baseline/N)

For discrimination, the best-performing models are for mortality, myocardial infarction, renal failure, and cerebrovascular accident, with high time-dependent AUC at 30 days (0.835, 0.802, 0.800, and 0.796, respectively) and high Uno’s concordance indices (0.845, 0.813, 0.805, and 0.802 respectively). The worst performing models are for readmission, venous thromboembolism, and sepsis, with Uno’s concordance indices of 0.617, 0.655, and 0.679, respectively. Uno’s and Harrell’s concordance indices are similar for all models.

All models are well-calibrated (S2(a) to S2(h) Figs in S3 Appendix), though the predictive ranges are limited to low probability areas due to the low incidence of outcomes. All models had low time-dependent Brier scores, scaled time-dependent Brier scores, and pseudo R-squares.

During sensitivity analysis, the performance measures of the exploratory accelerated failure time models were similar to those of the Cox proportional hazards models (S2.12 Table in S2 Appendix). To provide an example of time-to-event prediction, we plotted the risks over time for different postoperative outcomes for a theoretical patient (S4 Appendix). Analysis using AIC-based stepwise elimination for the sepsis and readmission models did not change alter model performance metrics (S5 Appendix).

## Discussion

The implementation of targeted, resource-efficient monitoring of postoperative complications requires accurate prediction of when a patient may be at highest risks of certain complications. In this NSQIP cohort of 132 145 patients, we developed and internally validated Cox proportional hazards models to predict time-to-event for major medical complications, readmission, and mortality within 30 days after elective colectomy. We found that while models for mortality, myocardial infarction, pneumonia, and renal failure performed well by concordance (> 0.8), the scaled Brier scores and pseudo R-squared values were poor. The models for readmission, venous thromboembolism, and sepsis performed poorly. All models displayed good calibration though with a limited range of prediction due to low incidence of complications.

Postoperative complications contribute to short- and long-term mortality, increased length of stay, readmission, and increased healthcare costs [4, 10–12].Since major complications have distinct temporal patterns [12, 20], accurate prediction of both if and when a complication may occur allows for targeted patient education and monitoring for specific complications at specific periods. We observed that the risk periods of each investigated complication differ, which has important implications for clinical management and targeted allocation of resources. Alarmingly, the median [quartiles] periods of diagnoses for most complications (ranging from 3 [1, 7] for myocardial infarction and 12 [6, 18] for venous thromboembolism) fall outside of the median [quartiles] for the length of stay of 4 [3, 6] days, highlighting the need for enhanced postoperative (and particularly post-discharge) monitoring, particularly after discharge.

Our time-to-event models add to the literature by facilitating efficient, targeted monitoring for complications in high risk patients during high risk periods. The NSQIP surgical risk calculator consists of 21 preoperative factors (demographics, comorbidities, and procedure) and was developed using 1 414 006 patients from 393 hospitals encompassing 1557 unique Current Procedural Terminology codes (i.e. procedures) [16]. However, its external validation in colectomy patients has been poor [18, 19]. The Codman score developed using a NSQIP dataset in South Africa is simpler with six predictors [17], with similar performances compared to the NSQIP morbidity and mortality algorithms when validated in 40 589 colectomy patients [19]. There are 26 models for elderly patients undergoing colorectal cancer surgery predicting outcomes, including mortality (n = 10), anastomotic leakage (n = 7), and surgical site infections (n = 3), with moderate to high bias in most studies [15]. Overall, the clinical utility of existing models is limited by the inability to predict the timing of complications for tailored monitoring strategies and prompt clinical intervention.

Among perioperative factors, postoperative complications have the greatest influence on both short-term and long-term mortality, compared to other preoperative and intraoperative determinants [10, 11]. Our study highlights the importance of incorporating temporality into postoperative risk prediction, providing a proof-of-concept of time-to-event risk prediction models that can be applied to other surgical populations to improve the detection and treatment of postoperative complications. Institutional perioperative data can be used to develop and continuously improve time-to-event postoperative risk prediction models [34]. This can guide informed consent with personalized risk calculators, disposition planning, and protocolized screening of postoperative complications. Importantly, since different complications tend to occur at different time ranges after surgery, spanning both during hospitalization and post-discharge, continuity of care and targeted monitoring are required to support patients. Hospital systems may leverage virtual care and remote monitoring for patients after discharge [35]. In a large randomized trial of patients undergoing noncardiac surgery, post-discharge remote monitoring did not increase the days alive and at home within 30 days of surgery [36]. However, there are signals of benefits for pain, reduced medication errors, and outcomes in centers with higher clinical response to the abnormalities detected [36]. Perioperative teams can use the graphs of risks for different complications over time (example in S4 Appendix) to educate patients about postoperative complications, identify patient-centered methods for self-screening, and empower patients to seek help across the risk periods.

There are several strengths in this study. The multicentered NSQIP dataset provides prospectively collected data that is likely generalizable to many other similar centers. There are clear definitions for each variable for the data collectors, with high inter-rater reliability in previous audits [37]. In terms of analysis, we performed time-to-event modelling, which provided additional temporal information compared to previous binary prediction models. Moreover, sensitivity analysis using accelerated failure time models was performed to ensure that the choice of modelling did not limit the model performances.

Our study has several limitations. First, there may have been measurement errors in the collected variables, particularly for the timing of the diagnosis of postoperative time-to-event outcomes. Some of the complications may be initially asymptomatic, and different patients experiencing complications may present with different levels of severity. Second, the severity of the outcomes was not available, but it is likely important for patients and clinicians to contextualize the predicted risk. Third, the NSQIP lacks intraoperative and many postoperative variables that may augment the models [38] and provide a real-time prediction that modifies risk prediction according to the patient’s postoperative course. Fourth, each model performance metric has limitations requiring nuanced interpretation [27]. The discrepancy between a high Harrell’s concordance index and low R-squared and scaled Brier scores may be due to low incidences of outcomes and a high percentage of censored data [32, 39]. The range of calibration was limited likely due to the low incidence of outcomes. Finally, the proportional hazard assumption was not satisfied for certain predictors. Nevertheless, the close correspondence in outcomes when comparing with results from bootstrapping and accelerated failure time models indicate that this did not significantly impact the results [33].

Future directions include validating the model using more recent NSQIP data and external validation using other prospective perioperative datasets, as well as optimizing predictors and modeling techniques. The incorporation of long-term outcome data would be important, as the 30-day mortality may underestimate the 90-day mortality rate [40]. Once refined and externally validated, the models could be incorporated into personalized risk calculators and postoperative screening guidelines. Finally, patients, caregivers, and clinicians should be engaged to optimize how risk information is conveyed for patient education and perioperative planning.

In conclusion, we developed and internally validated Cox proportional hazards models to predict time-to-event for major medical complications, readmission, and mortality elective colectomy. There are distinct temporal patterns for each postoperative complication. Once further validated, the models can facilitate tailored monitoring of high risk patients during high risk periods.

## Supporting information

S1 Appendix Definitions of variables. (DOCX)

S2 Appendix Supplemental tables. (DOCX)

S3 Appendix Supplemental figures. (DOCX)

S4 Appendix Example patient illustration of predictions. (DOCX)

S5 Appendix Sensitivity analysis for sepsis and readmission models. (DOCX)

## References

[1] Canadian Institute for Health Information. Surgeries Impacted by COVID-19, March 2020 to September 2022 –Data Tables. Ottawa, ON: CIHI; 2023.

[2] Healthcare Cost and Utilization Project (HCUP). Statistical Brief #281 Overview of Operating Room Procedures During Inpatient Stays in U.S. Hospitals, 2018. https://hcup-us.ahrq.gov/reports/statbriefs/sb281-Operating-Room-Procedures-During-Hospitalization-2018.jsp. Accessed November 24, 2023.

[3] Canadian Institute of Health Information. Inpatient Hospitalization, Surgery and Newborn Statistics, 2021–2022. Ottawa, ON: CIHI; 2023.

[4] Scarborough JE, Schumacher J, Kent KC, Heise CP, Greenberg CC. Associations of Specific Postoperative Complications With Outcomes After Elective Colon Resection: A Procedure-Targeted Approach Toward Surgical Quality Improvement. JAMA Surg 2017;152(2):e164681. doi: 10.1001/jamasurg.2016.4681 27926773

[5] Al-Mazrou AM, Onur B, Kiran RP. Failure of efforts to contain costs of care after colorectal procedures: Nationwide trends in length of stay, costs and post-acute care utilization. Am J Surg 2017;214(5):804–10. doi: 10.1016/j.amjsurg.2017.03.046 28473051

[6] Moloo H, Lacaille-Ranger A, MacLean A, Finley C. Pan-Canadian colorectal cancer surgery data: an opportunity for reflection and improvement. Can J Surg 2022;65(6):E735–8. doi: 10.1503/cjs.000621 36323443 PMC9633051

[7] Cram P, Cohen ME, Ko C, Landon BE, Hall B, Jackson TD. Surgical Outcomes in Canada and the United States: An Analysis of the ACS-NSQIP Clinical Registry. World J Surg 2022;46(5):1039–50. doi: 10.1007/s00268-022-06444-w 35102437 PMC9929717

[8] Al-Mazrou AM, Haiqing Z, Guanying Y, Kiran RP. Sustained positive impact of ACS-NSQIP program on outcomes after colorectal surgery over the last decade. Am J Surg 2020;219(1):197–205. doi: 10.1016/j.amjsurg.2019.05.001 31128841

[9] Zogg CK, Najjar P, Diaz AJR, Zogg DL, Tsai TC, Rose JA, et al. Rethinking Priorities: Cost of Complications After Elective Colectomy. Ann Surg 2016;264(2):312. doi: 10.1097/SLA.0000000000001511 26501705

[10] Khuri SF, Henderson WG, DePalma RG, Mosca C, Healey NA, Kumbhani DJ. Determinants of Long-Term Survival After Major Surgery and the Adverse Effect of Postoperative Complications. Ann Surg 2005;242(3):326–43. doi: 10.1097/01.sla.0000179621.33268.83 16135919 PMC1357741

[11] Silber JH, Rosenbaum PR, Trudeau ME, Chen W, Zhang X, Kelz RR, et al. Changes in prognosis after the first postoperative complication. Med Care 2005;43(2):122–31. doi: 10.1097/00005650-200502000-00005 15655425

[12] Morris MS, Deierhoi RJ, Richman JS, Altom LK, Hawn MT. The relationship between timing of surgical complications and hospital readmission. JAMA Surg 2014;149(4):348–54. doi: 10.1001/jamasurg.2013.4064 24522747

[13] McIsaac DI, MacDonald DB, Aucoin SD. Frailty for Perioperative Clinicians: A Narrative Review. Anesth Analg 2020;130(6):1450. doi: 10.1213/ANE.0000000000004602 32384334

[14] Hall KK, Lim A, Gale B: Chapter 2 Failure To Rescue, Making Healthcare Safer III: A Critical Analysis of Existing and Emerging Patient Safety Practices [Internet]. Edited by Hall KK, Shoemaker-Hunt S, Hoffman L, et al. Rockville, MD: Agency for Healthcare Research and Quality (US), 2020, pp 1–16. 32255576

[15] Souwer ETD, Bastiaannet E, Steyerberg EW, Dekker JWT, van den Bos F, Portielje JEA. Risk prediction models for postoperative outcomes of colorectal cancer surgery in the older population—a systematic review. J Geriatr Oncol 2020;11(8):1217–28. doi: 10.1016/j.jgo.2020.04.006 32414672

[16] Bilimoria KY, Liu Y, Paruch JL, Zhou L, Kmiecik TE, Ko CY, et al. Development and evaluation of the universal ACS NSQIP surgical risk calculator: a decision aid and informed consent tool for patients and surgeons. J Am Coll Surg 2013;217(5):833–842.e1-3. doi: 10.1016/j.jamcollsurg.2013.07.385 24055383 PMC3805776

[17] Spence RT, Chang DC, Kaafarani HMA, Panieri E, Anderson GA, Hutter MM. Derivation, Validation and Application of a Pragmatic Risk Prediction Index for Benchmarking of Surgical Outcomes. World J Surg 2018;42(2):533–40. doi: 10.1007/s00268-017-4177-2 28795214

[18] Hyde LZ, Valizadeh N, Al-Mazrou AM, Kiran RP. ACS-NSQIP risk calculator predicts cohort but not individual risk of complication following colorectal resection. Am J Surg 2019;218(1):131–5. doi: 10.1016/j.amjsurg.2018.11.017 30522696

[19] Spence RT, Guidolin K, Quereshy FA, Chadi SA, Chang DC, Hutter MM. External validation of the Codman score in colorectal surgery: a pragmatic tool to drive quality improvement. Colorectal Dis 2023;25(6):1248–56. doi: 10.1111/codi.16547 36965098

[20] Thompson JS, Baxter BT, Allison JG, Johnson FE, Lee KK, Park WY. Temporal patterns of postoperative complications. Arch Surg 2003;138(6):596–602; discussion 602–603. doi: 10.1001/archsurg.138.6.596 12799329

[21] American College of Surgeons. ACS NSQIP Participant Use Data File. https://www.facs.org/quality-programs/data-and-registries/acs-nsqip/participant-use-data-file. Accessed November 27, 2023.

[22] Collins GS, Reitsma JB, Altman DG, Moons KG. Transparent reporting of a multivariable prediction model for individual prognosis or diagnosis (TRIPOD): the TRIPOD Statement. BMC Medicine 2015;13(1):1. 25563062 10.1186/s12916-014-0241-z PMC4284921

[23] Riley RD, Snell KI, Ensor J, Burke DL, Harrell FE, Moons KG, et al. Minimum sample size for developing a multivariable prediction model: PART II—binary and time-to-event outcomes. Stat Med 2019;38(7):1276–96. doi: 10.1002/sim.7992 30357870 PMC6519266

[24] Austin PC, Harrell FE, Steyerberg EW. Predictive performance of machine and statistical learning methods: Impact of data-generating processes on external validity in the ‘large N, small p’ setting. Stat Methods Med Res. 2021;30(6):1465–83. doi: 10.1177/09622802211002867 33848231 PMC8188999

[25] Heinze G, Wallisch C, Dunkler D. Variable selection—A review and recommendations for the practicing statistician. Biom J 2018;60(3):431–49. doi: 10.1002/bimj.201700067 29292533 PMC5969114

[26] Parsa M, Keilegom IV. Accelerated failure time vs Cox proportional hazards mixture cure models: David vs Goliath? Statistical Papers 2023;64(3):835–55.

[27] Zhang Y, Wong G, Mann G, Muller S, Yang JYH. SurvBenchmark: comprehensive benchmarking study of survival analysis methods using both omics data and clinical data. Gigascience 2022;11:giac071. doi: 10.1093/gigascience/giac071 35906887 PMC9338425

[28] Hartman N, Kim S, He K, Kalbfleisch JD. Pitfalls of the concordance index for survival outcomes. Stat Med 2023;42(13):2179–90. doi: 10.1002/sim.9717 36977424 PMC10219847

[29] Longato E, Vettoretti M, Di Camillo B. A practical perspective on the concordance index for the evaluation and selection of prognostic time-to-event models. J Biomed Inform 2020;108:103496. doi: 10.1016/j.jbi.2020.103496 32652236

[30] Uno H, Cai T, Pencina MJ, D’Agostino RB, Wei LJ. On the C-statistics for evaluating overall adequacy of risk prediction procedures with censored survival data. Stat Med 2011;30(10):1105–17. doi: 10.1002/sim.4154 21484848 PMC3079915

[31] Graf E, Schmoor C, Sauerbrei W, Schumacher M. Assessment and comparison of prognostic classification schemes for survival data. Stat Med 1999;18(17–18):2529–45. doi: 10.1002/(sici)1097-0258(19990915/30)18:17/18&lt;2529::aid-sim274&gt;3.0.co;2-5 10474158

[32] Steyerberg EW, Vickers AJ, Cook NR, Gerds T, Gonen M, Obuchowski N, et al. Assessing the performance of prediction models: a framework for some traditional and novel measures. Epidemiology 2010;21(1):128–38. 20010215 10.1097/EDE.0b013e3181c30fb2 PMC3575184

[33] Stensrud MJ, Hernán MA. Why Test for Proportional Hazards? JAMA 2020;323(14):1401–2. doi: 10.1001/jama.2020.1267 32167523 PMC11983487

[34] Van Calster B, Steyerberg EW, Wynants L, van Smeden M. There is no such thing as a validated prediction model. BMC Med 2023;21(1):70. doi: 10.1186/s12916-023-02779-w 36829188 PMC9951847

[35] Leenen JPL, Ardesch V, Patijn G. Remote Home Monitoring of Continuous Vital Sign Measurements by Wearables in Patients Discharged After Colorectal Surgery: Observational Feasibility Study. JMIR Perioper Med. 2023;6:e45113. doi: 10.2196/45113 37145849 PMC10199380

[36] McGillion MH, Parlow J, Borges FK, Marcucci M, Jacka M, Adili A, et al. Post-discharge after surgery Virtual Care with Remote Automated Monitoring-1 (PVC-RAM-1) technology versus standard care: randomised controlled trial. BMJ. 2021;374:n2209. doi: 10.1136/bmj.n2209 34593374 PMC8477638

[37] Shiloach M, Frencher SK, Steeger JE, Rowell KS, Bartzokis K, Tomeh MG, et al. Toward robust information: data quality and inter-rater reliability in the American College of Surgeons National Surgical Quality Improvement Program. J Am Coll Surg. 2010;210(1):6–16. doi: 10.1016/j.jamcollsurg.2009.09.031 20123325

[38] Görges M, Afshar K, West N, Pi S, Bedford J, Whyte SD. Integrating intraoperative physiology data into outcome analysis for the ACS Pediatric National Surgical Quality Improvement Program. Paediatr Anaesth. 2019 Jan;29(1):27–37. doi: 10.1111/pan.13531 30347497

[39] Zhang Z. Semi-parametric regression model for survival data: graphical visualization with R. Ann Transl Med. 2016;4(23):461. doi: 10.21037/atm.2016.08.61 28090517 PMC5220043

[40] Visser BC, Keegan H, Martin M, Wren SM. Death after colectomy: it’s later than we think. Arch Surg. 2009;144(11):1021–7. doi: 10.1001/archsurg.2009.197 19917938

