# Supplementary material for: Development and internal validation of time-to-event risk prediction models for major medical complications within 30 days after elective colectomy
Source: PLoS One. 2024 Dec 2;19(12):e0314526. doi: 10.1371/journal.pone.0314526 (PMC11611139; doi:10.1371/journal.pone.0314526)
Supplement: S1 Appendix — (DOCX) [file pone.0314526.s001.docx]

**Appendix 1.** Definitions of Variables

NSQIP® contains more than 150 clinical variables within 30 days after surgery (1). This dataset includes information on whether the patient was diagnosed with major complications (in- or out-of-hospital) and the number of postoperative days to the diagnoses of complications, as defined by standardized criteria within the NSQIP operations manual. Please see Chapter 4 of “ACS NSQIP® Operations Manual” and Chapter 4 of “ACS NSQIP® Procedure Targeted Colectomy Variables and Definitions” for further details and definitions.

**S1.1 Table.** Variables Requested From NSQIP General Dataset (2014-2019).

| **NSQIP Variable Name** | **Variable definition and options** | **Function** |
| --- | --- | --- |
| OperYR | Year of surgery | Inclusion criteria  Cohort division |
| AGE | Age (character variable)    >90 is coded as 90+ in NSQIP  Created new variable **age_num** to set “90+” as 90 (numeric) and convert AGE to a numeric variable | Double check inclusion criteria of age >=18.    Predictor |
| ELECTSURG | 1 = elective  0 = emergent/urgent | Inclusion criteria |
| EMERGNCY | Yes; No; Unknown  (patients with “Yes”, “Unknown”, and missing values will be excluded unless ELECTSURG=1) | Exclusion criteria |
| VENTILAT | Patient requiring ventilator-assisted respiration at any time during the 48 hours preceding  the primary procedure  Yes; No | Exclusion criteria |
| DISCANCR | Disseminated cancer  Yes; No | Exclusion criteria |
| WNDINF | Open Wound (with or without Infection)  Yes; No | Exclusion criteria |
| PRSEPIS | Systemic Sepsis within 48 Hours Prior to Surgery  SIRS; Sepsis; Septic Shock; None  Note also need to exclude if SEPSISPATOS or SEPSHOCKPATOS are “Yes” | Exclusion criteria |
| ASACLAS | ASA Classification   - 1 -No Disturb - 2 -Mild Disturb - 3 -Severe Disturb - 4 -Life Threat - 5 -Moribund - None assigned | Exclusion criteria (ASACLAS = 5)  Predictor |
| HEIGHT | Height in inches  -99=No Response | Verify % missing of height and weight to determine data quality.  Calculate variable BMI, Body Mass Index (predictor) |
| WEIGHT | Weight in lbs  -99=No Response |  |
| SEX | Sex: Male; Female; non-binary  Note the dataset actually only has male vs. female | Predictor |
| RACE_NEW | Race  American Indian or Alaska Native  Asian  Black or African American  Native Hawaiian or Pacific Islander  White  Unknown/Not Reported | Predictor |
| DIABETES | Diabetes mellitus with oral agents or insulin  Insulin  No  Non-Insulin | Predictor |
| SMOKE | Current Smoker within One Year  Yes; No | Predictor |
| HXCOPD | History of severe chronic obstructive pulmonary disease  Yes; No | Predictor |
| DYSPNEA | Dyspnea  at rest; moderate exertion; no | Predictor |
| HXCHF | Congestive Heart Failure within 30 Days Prior to Surgery  Yes; No | Predictor |
| HYPERMED | Hypertension Requiring Medication  Yes; No | Predictor |
| ASCITES | Ascites within 30 Days Prior to Surgery  Yes; No | Predictor |
| BLEEDDIS | Bleeding disorders  Yes; No | Predictor |
| DIABETES | Diabetes Mellitus Requiring Therapy with Non-Insulin Agents or Insulin  No  Non-insulin  Insulin | Predictor |
| STEROID | Steroid/Immunosuppressant Use for a Chronic Condition  Yes; No | Predictor  If COL_STEROID=Yes, then STEROID=Yes (see Colectomy dataset below) |
| COL_CHEMO | Chemotherapy within 90 Days  Yes; No | Predictor |
| RENAFAIL  DIALYSIS  PRCREAT | Preoperative renal failure (**kidney**) will be defined as if yes to any of NSQIP variables Acute Renal Failure (RENAFAIL) OR Dialysis, OR GFR<= 60 (calculated by CKD-EPI formula using NSQIP preoperative creatinine, age, and gender. (2)     Derived from:   - Acute Renal Failure: RENAFAIL: Yes; No - Currently Requiring or On Dialysis: DIALYSIS: Yes; No - Pre-operative serum creatinine: PRCREAT | Predictor |
| FNSTATUS2 | Functional health status Prior to Surgery  Independent  Partially Dependent  Totally Dependent  Unknown | Predictor |
| OPTIME | Total operation time | Predictor |
| ANESTHES | Principal Anesthesia Technique  Epidural  General  Local  Monitored Anesthesia care (MAC) / IV Sedation None  Regional  Spinal  Other  Unknown | Combine info re. epidural, spinal info “Use of Regional Anesthesia” – i.e. if yes, then yes for the respective category for “Use of Regional Anesthesia” |
| ANESTHES_OTHER | Additional Anesthesia Technique(s)  General  Epidural  Spinal  Regional  Local  Monitored Anesthesia Care/IV Sedation Other |  |
| Use of Regional Anesthesia | Use of Regional Anesthesia  • Yes, thoracic epidural  • Yes, spinal anesthesia  • Yes, TAP blocks  • No: None of the above regional anesthesia methods were employed | Predictor |
| DOpertoD | Days from Operation to Death  -99= Patient did not die at or before 30 days post operation | To derive variable MORTALITY (30 days, all-cause):  Mortality30days = yes if DOpertoD not equal to -99 OR DOpertoD >30    Survival analysis |
| READMISSION1 | Hospital Readmission (at least 1)  Yes; No | Outcome |
| READMPODAYS1 | Days from principal operative procedure to first Readmission | Survival analysis |
| UNPLANNEDREADMISSION1 | Hospital Readmission - unplanned  Yes; No | Additional cohort characteristics |
| CDMI | Intraoperative or Postoperative Myocardial Infarction             Myocardial Infarction             No Complication | Outcome |
| DCDMI | Days from Operation until Myocardial Infarction Complication  -99 (i.e., missing) = Patient did not experience this complication at or before 30 days post operation (same for all Days from Operation variables below) | Survival analysis |
| CNSCVA | Stroke/Cerebral Vascular Accident (CVA) with neurological deficit             Stroke/CVA             No Complication | Outcome |
| DCNSCVA | Days from Operation until Stroke/CVA Complication | Survival analysis |
| OUPNEUMO | Occurrences Pneumonia  Pneumonia  No Complication | Outcome |
| PNAPATOS | Pneumonia – PATOS  Yes; No | Exclude from pneumonia outcome since existing preoperatively |
| DOUPNEUMO | Days from Operation until Pneumonia Complication | Survival analysis |
| OTHSYSEP | Sepsis  Sepsis; No Complication | To create composite outcome of SEPSIS = yes if sepsis or septic shock = yes)  Report separately from septic shock in additional cohort characteristics |
| SEPSISPATOS | Sepsis – PATOS  Yes; No | Exclude from sepsis outcome since existing preoperatively  Exclusion criteria of this study |
| DOTHSYSEP | Days from Operation until Sepsis Complication | Use the earliest of either DOTHSYSEP or DOTHSESHOCK for the “Days from Operation until sepsis or septic shock” variable (DSEPSIS)  Survival analysis |
| OTHSESHOCK | Septic Shock  Septic Shock; No Complication | To create composite outcome of SEPSIS = yes if sepsis or septic shock = yes)  Report separately from septic shock in additional cohort characteristics |
| SEPSHOCKPATOS | Septic Shock PATOS  Yes; No | Exclude from septic shock outcome since existing preoperatively  Exclusion criteria of this study |
| DOTHSESHOCK | Days from Operation until Septic Shock Complication | Use the earliest of either DOTHSYSEP or DOTHSESHOCK for the “Days from Operation until sepsis or septic shock” variable (DSEPSIS)  Survival analysis |
| OPRENAFL | Progressive Renal Insufficiency/Acute Renal Failure Requiring Dialysis  Acute Renal Failure; No Complication | Outcome |
| DOPRENAFL | Days from Operation until Acute Renal Failure Complication | Survival analysis |
| PULEMBOL | Occurrences Pulmonary Embolism  Pulmonary Embolism; No Complication | To derive outcome variable VTE (Venous thromboembolism)  Yes = either PULEMBOL = Pulmonary Embolism OR OTHDVT = DVT Requiring Therapy    Report separately in additional cohort characteristics |
| OTHDVT | Occurrences DVT  DVT Requiring Therapy  No Complication |  |
| DPULEMBOL | Days from Operation until Pulmonary Embolism Complication | Use the earliest of either DPULEMBOL or DOTHDVT for the “Days from Operation until VTE” variable (DVTE)    Survival analysis |
| DOTHDVT | Days from Operation until DVT/Thrombophlebitis Complication |  |
| URNINFEC | Urinary Tract Infection  Urinary Tract Infection; No Complication | Additional cohort characteristics |
| UTIPATOS | UTI PATOS  Yes; No | Exclude from UTI variable since existing preoperatively |
| DOptoDis | Length of postoperative hospital stay: Days from operation to discharge | Additional cohort characteristics |
| DISCHDEST | Discharge Destination  Home  Skilled Care, Not Home  Unskilled Facility Not Home  Facility Which was Home  Separate Acute Care  Rehab  Expired  Against Medical Advice (AMA)  Multi - level Senior Community Hospice  Unknown | For additional cohort characteristics.  Create new variable of NONHOME = no if DISCHDEST  = home, Facility Which was Home, or AMA; else NONHOME = yes) |
| FAILWEAN | On Ventilator > 48 Hours  On Ventilator greater than 48 Hours; No Complication | Additional cohort characteristics |
| VENTPATOS | On Ventilator > 48 Hours – PATOS  Yes; No | Exclude from ventilator > 48 hours variable since existing preoperatively  Exclusion criteria |
| CDARREST | Intraoperative or Postoperative Cardiac Arrest Requiring CPR  Cardiac Arrest Requiring CPR; No Complication | Additional cohort characteristics |
| DCDARREST | Days from Operation until Cardiac Arrest Requiring CPR Complication | Additional cohort characteristics |
| OTHBLEED | Transfusion Intra/Postop (RBC within the First 72 Hrs of Surgery Start Time)  Transfusions/Intraop/Postop; No Complication | Additional cohort characteristics |
| DOTHBLEED | Days from Operation until Bleeding Transfusions Complication | Additional cohort characteristics |
| STILLINHOSP | Still in Hospital > 30 Days  Yes; No | Additional cohort characteristics |
| REOPERATION1 | Unplanned Reoperation (at least 1)  Yes; No | Additional cohort characteristics |
| WNDCLAS | Wound Classification  1-Clean  2-Clean/Contaminated   3-Contaminated   4-Dirty/Infected | Additional cohort characteristics  Display 1 and 2 in the same category per Scarborough et al. |

PATOS = present on admission.

**S1.2 Table.** Variables requested from NSQIP Colectomy Procedure Targeted Dataset (2014-2019).

| Variable name | Legend | Reason for request |
| --- | --- | --- |
| IDN | NSQIP ID (deidentified) | To link ID the general and Colectomy datasets  Inclusion |
| COL_STEROID | Steroid/Immunosuppressant Use for Inflammatory Bowel Disease  Yes; No; Unknown/NULL | Combine with STEROID above |
| COL_STEROID_UNK | Corresponds to the NAs above | Not needed |
| COL_INDICATION | Primary Indication for Surgery:  Acute diverticulitis  Bleeding  Chronic diverticular disease  Colon cancer  Colon cancer w/ obstruction  Crohn's Disease  Enterocolitis (e.g. C. Difficile)  NULL=missing  Non-malignant polyp  Other-Enter ICD-10 for diagnosis  Ulcerative colitis  Unknown  Volvulus    **New categorical predictor variable to be created from above info** (3)  **INDICATION:**  Cancer = Colon cancer; Colon cancer w/ obstruction  IBD (Inflammatory bowel disease) = Crohn's Disease, Ulcerative colitis  Diverticulitis = Chronic diverticular disease)  Non-malignant polyp  Bleeding  Other  Missing = NULL, unknown | Full list reported in cohort characteristics    Exclude Acute diverticulitis, Enterocolitis (e.g. C. Difficile), and Volvulus due to non-elective    Recode into a predictor |
| COL_APPROACH | Operative approach:  Endoscopic  Endoscopic w/ open assist  Endoscopic w/ unplanned conversion to open  Hybrid  Hybrid w/ open assist  Hybrid w/ unplanned conversion to open  Laparoscopic  Laparoscopic w/ open assist  Laparoscopic w/ unplanned conversion to open  NOTES w/ open assist  Open (planned)  Other  Other MIS approach  Other MIS approach w/ open assist  Robotic  Robotic w/ open assist  Robotic w/ unplanned conversion to open  SILS (single incision laparoscopic surgery)  SILS w/ open assist  SILS w/ unplanned conversion to open  Unknown    **New predictor to be created from above info** (3)  **APPROACH**:  Open = Open (planned)    Minimally invasive = Endoscopic, Laparoscopic, NOTES, Other MIS approach, Robotic    Minimally invasive with open- or hand-assist = Endoscopic w/ open assist, Hybrid, Hybrid w/ open assist, Laparoscopic w/ open assist, NOTES w/ open assist, Other MIS approach w/ open assist, Robotic w/ open assist, SILS, SILS w/ open assist    Minimally invasive with unplanned conversion to open = Endoscopic w/ unplanned conversion to open, Hybrid w/ unplanned conversion to open, Laparoscopic w/ unplanned conversion to open, NOTES w/ unplanned conversion to open, Other MIS approach w/ unplanned conversion to open, Robotic w/ unplanned conversion to open, SILS w/ unplanned conversion to open    Other/unknown = Other, Unknown | Full list reported in cohort characteristics |
| COL_ANASTOMOTIC | Anastomotic Leak:  Leak, no treatment intervention documented  Leak, treated w/ interventional means  Leak, treated w/ non-interventional/non-operative means  Leak, treated w/ reoperation  No definitive diagnosis of leak/leak related abscess  Unknown | Additional cohort characteristics |
| COL_ILEUS | Prolonged Postoperative NPO or NGT Use | Additional cohort characteristics |
| COL_ILEUS_UNK | Corresponds to the NAs above | Not needed |
| COL_CHEMO | Chemotherapy within 90 Days  Yes; No | Predictor |
| COL_CHEMO_UNK | Corresponds to the NAs above | Not needed |
| COL_ICD10_INDICATION | ICD10 for Indication  Yes; No |  |

Missing was coded in NSQIP as -99, unknown, and NULL. Each variable was individually examined to ensure that the missing value was correctly standardized.  **S1.3 Table.** New Variables Created During Data Processing.

| **Variable name** | **Legend** | **Comments** |
| --- | --- | --- |
| **age_num** | Age in numeric | 90+ from Age (character variable) set to be 90 |
| **BMI** | col_all2$BMI <- (col_all2$WEIGHT / (col_all2$HEIGHT)^2) * 703 | #note units are lb and inches in NSQIP  #formula https://www.cdc.gov/healthyweight/assessing/bmi/childrens_bmi/childrens_bmi_formula.html |
| **KIDNEY** | Preoperative renal failure (**KIDNEY**) was defined as if yes to any of NSQIP variables Acute Renal Failure (RENAFAIL) OR Dialysis, OR GFR<= 60 (calculated by CKD-EPI formula using NSQIP preoperative creatinine, age, and gender (2).  Derived from:   - Acute Renal Failure: RENAFAIL: Yes; No - Currently Requiring or On Dialysis: DIALYSIS: Yes; No - Pre-operative serum creatinine: PRCREAT   SAS codes from previous project - to adapt to R  /* Renal failure */  If sex='female' then female=1;  If sex='male' then female=0;    creatinine=.;  If PRCREAT gt 0 then creatinine=PRCREAT;    * GFR http://nephron.com/epi_equation https://www.reddit.com/r/sas/comments/3id5kx/need_some_help_modeling_an_equation/;    if female=0 then kappa = 0.9;  else if female=1 then kappa = 0.7;    if female=0 then alpha = -0.411;  else if female=1 then alpha = -0.329;    if female=1 then constant1 = 1.018;  else constant1 = 1;    if RACE_NEW = 'Black or African American' then constant2 = 1.159;  else constant2 = 1;    GFR = 141 * (MIN(creatinine / kappa, 1) ** alpha) * (MAX(creatinine / kappa, 1) ** (-1.209)) * (0.993 ** new_age) * constant1 * constant2;    If DIALYSIS='No' OR RENAFAIL='No' then RF_pre=0;  If GFR lt 60 then RF_pre=1;  If DIALYSIS='Yes' OR RENAFAIL='Yes' then RF_pre=1; | |
| **MORTALITY** | (30 days, all-cause):  Mortality30days = yes if DOpertoD not equal to -99 OR DOpertoD >30 |  |
| **PNA_NEW** | Yes: OUPNEUMO = Pneumonia AND PNAPATOS = No  No = OUPNEUMO = No Complication or  (OUPNEUMO = Pneumonia AND PNAPATOS = Yes) |  |
| **SEPSIS** | **SEPSIS** = Yes if OTHSYSEP = Sepsis OR OTHSESHOCK= Septic Shock, else No |  |
| **~~SEPSIS_NEW~~** | Yes: SEPSIS = Yes AND SEPSISPATOS = No AND SEPSHOCKPATOS = No    Else No | Note that in our dataset, SEPSI = SEPSIS_NEW since we excluded pre-existing sepsis. Thus this variable was not created |
| **DSEPSIS** | Days from Operation until Septic Shock OR sepsis Complication: minimum of the DOTHSYSEP or DOTHSESHOCK |  |
| **~~DSEPSIS_NEW~~** | Days from Operation until Septic Shock OR sepsis Complication:  minimum of the DOTHSYSEP or DOTHSESHOCK | Note that in our dataset, SEPSIS = SEPSIS_NEW since we excluded pre-existing sepsis (PATOS). Thus this variable was not created |
| **VTE** | Venous thromboembolism  Yes = either PULEMBOL = Pulmonary Embolism OR OTHDVT = DVT Requiring Therapy  Else No |  |
| **DVTE** | Days from Operation until VTE:  Minimum of DPULEMBOL or DOTHDVT |  |
| **UTI_NEW** | Yes:  URNINFEC = Urinary Tract Infection AND  UTIPATOS  = No  No =  URNINFEC = No Complication or  (URNINFEC =  Urinary Tract Infection AND UTIPATOS = Yes) |  |
| **NONHOME** | **NONHOME** = no if DISCHDEST  = Home, Facility Which was Home, or  Against Medical Advice (AMA); else NONHOME = yes) |  |
| **EPIDURAL** | Yes, No    col_all2$EPIDURAL <- ifelse(col_all2$ANESTHES=="Epidural" \| col_all2$ANESTHES_OTHER=="Epidural" \| col_all2$ANESTHES_OTHER=="Epidural,Local" \| col_all2$ANESTHES_OTHER=="Epidural,Local,Other" \| col_all2$ANESTHES_OTHER=="Epidural,Local,Monitored Anesthesia Care/IV Sedation" \| col_all2$ANESTHES_OTHER=="Epidural,Monitored anesthesia care/IV sedation" \| col_all2$ANESTHES_OTHER=="Epidural,Monitored Anesthesia Care/IV Sedation" \| col_all2$ANESTHES_OTHER=="Epidural,Other" \| col_all2$ANESTHES_OTHER=="Epidural,Regional" \| col_all2$ANESTHES_OTHER=="Epidural,Regional,Local" \| col_all2$ANESTHES_OTHER=="Epidural,Regional,Other" \| col_all2$ANESTHES_OTHER=="Epidural,Spinal" \| col_all2$ANESTHES_OTHER=="Epidural,Spinal,Local" \| col_all2$ANESTHES_OTHER=="Epidural,Spinal,Regional" \| col_all2$ANESTHES_OTHER=="General,Epidural" \| col_all2$ANESTHES_OTHER=="General,Epidural,Regional" \| col_all2$ANESTHES_OTHER=="Spinal,Epidural" \| col_all2$ANESTHES_OTHER=="Spinal,Epidural,Local" \| col_all2$ANESTHES_OTHER=="Spinal,Epidural,Regional",                              "Yes", "No")    #assume NA = no epidural, set NA as No  col_all2$EPIDURAL <- ifelse(is.na(col_all2$EPIDURAL), "No", col_all2$EPIDURAL)  sum(is.na(col_all2$EPIDURAL)) | |
| **SPINAL** | Yes, No  col_all2$SPINAL <- ifelse(col_all2$ANESTHES=="Spinal" \| col_all2$ANESTHES_OTHER=="Epidural,Spinal" \| col_all2$ANESTHES_OTHER=="Epidural,Spinal,Local" \| col_all2$ANESTHES_OTHER==" Epidural,Spinal,Regional" \| col_all2$ANESTHES_OTHER=="General,Spinal" \| col_all2$ANESTHES_OTHER=="General,Spinal,Local" \| col_all2$ANESTHES_OTHER=="General,Spinal,Regional" \| col_all2$ANESTHES_OTHER=="Spinal" \| col_all2$ANESTHES_OTHER=="Spinal,Epidural" \| col_all2$ANESTHES_OTHER=="Spinal,Epidural,Local" \| col_all2$ANESTHES_OTHER=="Spinal,Epidural,Regional" \| col_all2$ANESTHES_OTHER=="Spinal,Local" \| col_all2$ANESTHES_OTHER=="Spinal,Local,Monitored Anesthesia Care/IV Sedation" \| col_all2$ANESTHES_OTHER=="Spinal,Monitored anesthesia care/IV sedation" \| col_all2$ANESTHES_OTHER=="Spinal,Monitored Anesthesia Care/IV Sedation" \| col_all2$ANESTHES_OTHER=="Spinal,Other" \| col_all2$ANESTHES_OTHER=="Spinal,Regional" \| col_all2$ANESTHES_OTHER=="Spinal,Regional,Local" \| col_all2$ANESTHES_OTHER=="Spinal,Regional,Monitored anesthesia care/IV sedation" \| col_all2$ANESTHES_OTHER=="Spinal,Regional,Other",                              "Yes", "No")    #assume NA = no, set NA as No  col_all2$SPINAL <- ifelse(is.na(col_all2$SPINAL), "No", col_all2$SPINAL) | |
| **REGIONAL** | Yes, No  col_all2$REGIONAL <- ifelse(col_all2$ANESTHES=="Regional" \| col_all2$ANESTHES_OTHER=="Epidural,Regional" \| col_all2$ANESTHES_OTHER=="Epidural,Regional,Local" \| col_all2$ANESTHES_OTHER=="Epidural,Regional,Other" \| col_all2$ANESTHES_OTHER=="Epidural,Spinal,Regional" \| col_all2$ANESTHES_OTHER=="General,Epidural,Regional" \| col_all2$ANESTHES_OTHER=="General,Regional" \| col_all2$ANESTHES_OTHER=="General,Regional,Local" \| col_all2$ANESTHES_OTHER=="General,Spinal,Regional" \| col_all2$ANESTHES_OTHER=="Regional" \| col_all2$ANESTHES_OTHER=="Regional,Local" \| col_all2$ANESTHES_OTHER=="Regional,Local,Monitored Anesthesia Care/IV Sedation" \| col_all2$ANESTHES_OTHER=="Regional,Local,Other" \| col_all2$ANESTHES_OTHER=="Regional,Monitored anesthesia care/IV sedation" \| col_all2$ANESTHES_OTHER=="Regional,Monitored Anesthesia Care/IV Sedation" \| col_all2$ANESTHES_OTHER=="Regional,Other" \| col_all2$ANESTHES_OTHER=="Spinal,Epidural,Regional" \| col_all2$ANESTHES_OTHER=="Spinal,Regional" \| col_all2$ANESTHES_OTHER=="Spinal,Regional,Local" \| col_all2$ANESTHES_OTHER=="Spinal,Regional,Monitored anesthesia care/IV sedation" \| col_all2$ANESTHES_OTHER=="Spinal,Regional,Other","Yes", "No")  table(col_all2$REGIONAL)    #assume NA = no, set NA as No  col_all2$REGIONAL <- ifelse(is.na(col_all2$REGIONAL), "No", col_all2$REGIONAL) | |
| **INDICATION** | **New categorical predictor variable to be created from COL_INDICATION** (per Scarborough et. al.)  **INDICATION:**  Cancer = Colon cancer; Colon cancer w/ obstruction  IBD (Inflammatory bowel disease) = Crohn's Disease, Ulcerative colitis  Diverticulitis = Chronic diverticular disease  Non-malignant polyp  Bleeding  Other = Other-Enter ICD-10 for diagnosis  NA | Excluded Acute diverticulitis, Enterocolitis (e.g. C. Difficile), and Volvulus due to non-elective |
| **APPROACH** | (Planned approach)    **New predictor to be created from  COL_APPROACH** (per Scarborough et al.)  **APPROACH**:    Open = Open (planned)    Minimally invasive = Endoscopic, Laparoscopic, NOTES, Other MIS approach, Robotic      Minimally invasive with open- or hand-assist = Endoscopic w/ open assist, Hybrid, Hybrid w/ open assist, Laparoscopic w/ open assist, NOTES w/ open assist, Other MIS approach w/ open assist, Robotic w/ open assist, SILS, SILS w/ open assist    Minimally invasive with unplanned conversion to open = Endoscopic w/ unplanned conversion to open, Hybrid w/ unplanned conversion to open, Laparoscopic w/ unplanned conversion to open,  NOTES w/ unplanned conversion to open,  Other MIS approach w/ unplanned conversion to open, Robotic w/ unplanned conversion to open, SILS w/ unplanned conversion to open    Other = Other    Unknown already converted NAs | Full list reported in cohort characteristics |
| **STEROID_NEW** | col_all2$STEROID_NEW<- ifelse(col_all2$COL_STEROID == "Yes" \| col_all2$STEROID == "Yes", "Yes", "No") | Two variables for steroid in NSQIP |

**References**

1. ACS NSQIP Participant Use Data File [Internet]. American College of Surgeons. [cited 2021 Sep 18]. Available from: http://www.facs.org/quality-programs/acs-nsqip/participant-use

2. Levey AS, Stevens LA, Schmid CH, Zhang YL, Castro AF, Feldman HI, et al. A new equation to estimate glomerular filtration rate. Ann Intern Med. 2009 May 5;150(9):604–12.

3. Scarborough JE, Schumacher J, Kent KC, Heise CP, Greenberg CC. Associations of Specific Postoperative Complications With Outcomes After Elective Colon Resection: A Procedure-Targeted Approach Toward Surgical Quality Improvement. JAMA Surgery. 2017 Feb 15;152(2):e164681.
