# Supplementary material for: Development and internal validation of time-to-event risk prediction models for major medical complications within 30 days after elective colectomy
Source: PLoS One. 2024 Dec 2;19(12):e0314526. doi: 10.1371/journal.pone.0314526 (PMC11611139; doi:10.1371/journal.pone.0314526)
Supplement: S2 Appendix — (DOCX) [file pone.0314526.s002.docx]

**Appendix 2.** Supplemental Tables.

**S2.1 Table.** Incidence and Timeline of Postoperative Morbidity and Mortality.

| **Outcome** | **Postoperative Day of Event** | **Incidence** |
| --- | --- | --- |
|  | **Median (IQR)** | **N (%)** |
| **Bleeding requiring transfusion within 72 hours after start of surgery** | 1 (0-2) | 6986 (5.3) |
| **Myocardial infarction** | 3 (1-7) | 650 (0.5) |
| **Pneumonia** | 5 (3-10) | 1444 (1.1) |
| **Cardiac arrest** | 6 (2-10) | 383 (0.3) |
| **Cerebrovascular event** | 7 (2-14) | 223 (0.2) |
| **Sepsis or septic shock** | 8 (5-13) | 3392 (2.6) |
| **Acute renal failure** | 9 (4-16) | 358 (0.3) |
| **Mortality** | 10 (6-18) | 714 (0.5) |
| **Venous thromboembolism** | 12 (6-18) | 1523 (1.2) |
| **Readmission** | 12 (8-19) | 11 415 (8.6) |
| **Reoperation** | N/A | 4873 (3.7) |
| **Still in hospital postoperative day 30** | N/A | 608 (0.5) |
| **Discharge destination not being preoperative residence** | N/A | 6135 (4.6) |
| **Urinary tract infectison** | N/A | 2017 (1.5) |
| **Anastomatic leak requiring treatment** | N/A | 3245 (2.5) |
| **Prolonged ileus** | N/A | 14 288 (10.8) |
| **On ventilator > 48 hours postoperatively** | N/A | 751 (0.6) |

Incidences of postoperative morbidity and mortality, listed according to the postoperative day of occurrence. N/A = data not available. The size of the total cohort is 132 145. Patients with missing values are assumed to not have had the complication.

**S2.2 Table.** Predictors That Did Not Meet Assumptions of Cox Proportional-Hazard Models.

| **Model Endpoint** | **Assumptions** | **Predictors that did not meet assumptions** |
| --- | --- | --- |
| **Mortality** | Proportional hazards | ASA PS, BMI, primary Indication for colectomy |
|  | Linearity | None |
| **Myocardial infarction** | Proportional hazards | Preoperative renal failure |
|  | Linearity | None |
| **Acute renal failure** | Proportional hazards | Operative approach |
|  | Linearity | None |
| **Cerebrovascular event** | Proportional hazards | Any regional anesthesia use |
|  | Linearity | None |
| **Pneumonia** | Proportional hazards | Age, smoker within one year preoperatively |
|  | Linearity | None |
| **Sepsis** | Proportional hazards | ASA PS, BMI, congestive heart failure within 30 days preoperatively, hypertension requiring medication, bleeding disorders or on anticoagulants, chemotherapy within 90 days preoperatively, primary indication for colectomy, total operation time, wound classification |
|  | Linearity | Total operation time |
| **Venous thromboembolism** | Proportional hazards | ASA PS, BMI, Operative approach, total operation time, wound classification |
|  | Linearity | Total operation time |
| **Readmission** | Proportional hazards | Age, Sex, ASA PS, BMI, diabetes, history of severe COPD, congestive heart failure within 30 days preoperatively, preoperative renal failure, hypertension requiring medication, preoperative functional health status, dyspnea, bleeding disorders or on anticoagulants, steroid/immunosuppressant use for a chronic condition, chemotherapy within 90 days preoperatively, ascites within 30 days preoperatively, primary indication for colectomy, operative approach, total operation time, wound classification, any regional anesthesia use |
|  | Linearity | Total operation time |

ASA PS, American Society of Anesthesiologists Physical Status; BMI, body mass index; COPD, chronic obstructive pulmonary disease.

**S2.3 Table.** The Features and Coefficients for the Cox Proportional-Hazard Model for Readmission.

|  | **Cox PH** | | | **AFT** | | |
| --- | --- | --- | --- | --- | --- | --- |
| **Predictor** | **Coefficient** | **SE** | **p-value** | **Coefficient** | **SE** | **p-value** |
| Intercept |  |  |  | 5.6288 | 0.1943 | <0.001 |
| Age | -0.0022 | < 0.001 | 0.0106 | 0.0019 | < 0.001 | 0.0103 |
| Male sex | -0.0096 | 0.0192 | 0.6183 | 0.009 | 0.0164 | 0.5827 |
| ASA PS |  |  |  |  |  |  |
| I (Reference) |  |  |  |  |  |  |
| II | 0.0307 | 0.0811 | 0.7052 | -0.0262 | 0.069 | 0.7043 |
| III | 0.2727 | 0.0819 | 0.0009 | -0.2335 | 0.0697 | 0.0008 |
| IV | 0.4846 | 0.0924 | <0.001 | -0.416 | 0.0787 | <0.001 |
| Body Mass Index | -0.0028 | 0.0015 | 0.0664 | 0.0024 | 0.0013 | 0.0638 |
| Diabetes |  |  |  |  |  |  |
| Insulin | 0.1934 | 0.04 | <0.001 | -0.1675 | 0.0341 | <0.001 |
| Non-Insulin | 0.0927 | 0.0303 | 0.0022 | -0.0805 | 0.0257 | 0.0018 |
| History of severe COPD | 0.1777 | 0.0428 | <0.001 | -0.153 | 0.0364 | <0.001 |
| Smoker within one year preoperatively | 0.1709 | 0.0254 | <0.001 | -0.1459 | 0.0216 | <0.001 |
| Congestive heart failure, within 30 days before surgery | 0.3165 | 0.0945 | 0.0008 | -0.2743 | 0.0804 | 0.0006 |
| Preoperative renal failure (preoperative acute renal failure, dialysis, and/or GFR ≤ 60 mL/min) | 0.1688 | 0.0274 | <0.001 | -0.1451 | 0.0234 | <0.001 |
| Hypertension requiring medication | 0.1216 | 0.0222 | <0.001 | -0.1042 | 0.0189 | <0.001 |
| Preoperative functional health status |  |  |  |  |  |  |
| Independent (Reference) |  |  |  |  |  |  |
| Partially dependent | 0.168 | 0.0782 | 0.0318 | -0.1441 | 0.0665 | 0.0303 |
| Totally dependent | 0.7515 | 0.175 | <0.001 | -0.6533 | 0.1489 | <0.001 |
| Dyspnea |  |  |  |  |  |  |
| At rest | 0.1349 | 0.1613 | 0.4029 | -0.1115 | 0.1372 | 0.4166 |
| Moderate exertion | 0.0614 | 0.0388 | 0.1133 | -0.0525 | 0.033 | 0.1118 |
| Bleeding disorders or on anticoagulants | 0.3396 | 0.0547 | <0.001 | -0.2921 | 0.0466 | <0.001 |
| Steroid/immunosuppressant use for a chronic condition | 0.2328 | 0.0367 | <0.001 | -0.2002 | 0.0313 | <0.001 |
| Chemotherapy within 90 days preoperatively | 0.4123 | 0.0356 | <0.001 | -0.355 | 0.0304 | <0.001 |
| Ascites within 30 days preoperatively | 0.3025 | 0.1834 | 0.0990 | -0.2677 | 0.1559 | 0.0860 |
| Primary indication for colectomy |  |  |  |  |  |  |
| Bleeding (Reference) |  |  |  |  |  |  |
| Cancer | -0.5262 | 0.1777 | 0.0031 | 0.459 | 0.1511 | 0.0024 |
| Diverticulitis | -0.635 | 0.179 | 0.0004 | 0.5516 | 0.1522 | 0.0003 |
| IBD | -0.3371 | 0.1821 | 0.0641 | 0.2973 | 0.1548 | 0.0548 |
| Non-malignant polyp | -0.5531 | 0.1797 | 0.0021 | 0.4824 | 0.1529 | 0.0016 |
| Other | -0.3858 | 0.1788 | 0.0309 | 0.3388 | 0.152 | 0.0258 |
| Operative approach |  |  |  |  |  |  |
| Minimally invasive (Reference) |  |  |  |  |  |  |
| Minimally invasive with open- or hand-assist | 0.0998 | 0.0235 | <0.001 | -0.0853 | 0.02 | <0.001 |
| Minimally invasive with unplanned conversion to open | 0.3863 | 0.0346 | <0.001 | -0.3321 | 0.0296 | <0.001 |
| Open | 0.3632 | 0.0257 | <0.001 | -0.3129 | 0.022 | <0.001 |
| Other | -0.1336 | 0.4476 | 0.7652 | 0.1149 | 0.3806 | 0.7628 |
| Total operation time | 0.0015 | < 0.001 | <0.001 | -0.0013 | < 0.001 | <0.001 |
| Wound classification |  |  |  |  |  |  |
| Clean (Reference) |  |  |  |  |  |  |
| Clean/contaminated | 0.0637 | 0.1017 | 0.5312 | -0.0555 | 0.0864 | 0.5211 |
| Contaminated | 0.1562 | 0.1048 | 0.1359 | -0.1351 | 0.0891 | 0.1295 |
| Dirty/infected | 0.1814 | 0.1091 | 0.0962 | -0.1572 | 0.0928 | 0.0901 |
| Any regional anesthesia use | -0.0081 | 0.0229 | 0.7239 | 0.0066 | 0.0195 | 0.7342 |
|  |  |  |  |  |  |  |

ASA PS, American Society of Anesthesiologists Physical Status; COPD, chronic obstructive pulmonary disease; GFR, glomerular filtration rate as calculated using Chronic Kidney Disease Epidemiology Collaboration (CKD-EPI) formula; IBD, Inflammatory bowel disease; IQR, interquartile range; SE, standard error.

**S2.4 Table.** The Features And Coefficients for the Cox Proportional-Hazard Model for Mortality**.**

|  | **Cox PH** | | | **AFT** | | |
| --- | --- | --- | --- | --- | --- | --- |
| **Feature** | **Coefficient** | **SE** | **p-value** | **Coefficient** | **SE** | **p-value** |
| Intercept |  |  |  | 14.4432 | 1.0914 | <0.001 |
| Age | 0.064 | 0.0044 | <0.001 | -0.0693 | 0.0054 | <0.001 |
| Male sex | 0.4642 | 0.079 | <0.001 | -0.5016 | 0.0875 | <0.001 |
| ASA PS |  |  |  |  |  |  |
| I (Reference) |  |  |  |  |  |  |
| II | -0.2091 | 0.5885 | 0.7223 | 0.2262 | 0.6363 | 0.7222 |
| III | 0.5523 | 0.5848 | 0.3449 | -0.5971 | 0.6327 | 0.3453 |
| IV | 1.2418 | 0.5928 | 0.0362 | -1.3436 | 0.6429 | 0.0366 |
| Body Mass Index | -0.0149 | 0.0067 | 0.0253 | 0.0162 | 0.0072 | 0.0253 |
| Diabetes |  |  |  |  |  |  |
| Insulin | 0.0301 | 0.1435 | 0.8336 | -0.0345 | 0.1551 | 0.8240 |
| Non-Insulin | -0.0167 | 0.1089 | 0.8778 | 0.0178 | 0.1177 | 0.8797 |
| History of severe COPD | 0.3395 | 0.12 | 0.0047 | -0.3697 | 0.1305 | 0.0046 |
| Smoker within one year preoperatively | 0.3081 | 0.1147 | 0.0072 | -0.3333 | 0.1246 | 0.0075 |
| Congestive heart failure, within 30 days before surgery | 0.8852 | 0.1763 | <0.001 | -0.9575 | 0.1939 | <0.001 |
| Preoperative renal failure (preoperative acute renal failure, dialysis, and/or GFR ≤ 60mL/min) | 0.0644 | 0.0876 | 0.4618 | -0.0687 | 0.0947 | 0.4682 |
| Hypertension requiring medication | 0.1898 | 0.0921 | 0.0393 | -0.2049 | 0.0999 | 0.0402 |
| Preoperative functional health status |  |  |  |  |  |  |
| Independent (Reference) |  |  |  |  |  |  |
| Partially dependent | 0.8830 | 0.1508 | <0.001 | -0.9545 | 0.1669 | <0.001 |
| Totally dependent | 1.7665 | 0.3224 | <0.001 | -1.9206 | 0.3557 | <0.001 |
| Dyspnea |  |  |  |  |  |  |
| At rest | 0.4610 | 0.3463 | 0.1831 | -0.4857 | 0.375 | 0.1953 |
| Moderate exertion | 0.3481 | 0.1093 | 0.0015 | -0.377 | 0.1191 | 0.0016 |
| Bleeding disorders or on anticoagulants | 0.2375 | 0.1688 | 0.1596 | -0.2581 | 0.1828 | 0.1579 |
| Steroid/immunosuppressant use for a chronic condition | 0.3220 | 0.1483 | 0.0299 | -0.3496 | 0.1609 | 0.0298 |
| Chemotherapy within 90 days preoperatively | -0.2949 | 0.1956 | 0.1315 | 0.3197 | 0.2118 | 0.1312 |
| Ascites within 30 days preoperatively | 1.5002 | 0.3079 | <0.001 | -1.6218 | 0.338 | <0.001 |
| Primary indication for colectomy |  |  |  |  |  |  |
| Bleeding (Reference) |  |  |  |  |  |  |
| Cancer | -0.0045 | 0.584 | 0.9939 | 0.0024 | 0.6316 | 0.9970 |
| Diverticulitis | -0.9065 | 0.6064 | 0.1350 | 0.9783 | 0.6568 | 0.1363 |
| IBD | -0.5580 | 0.6472 | 0.3886 | 0.6026 | 0.7003 | 0.3895 |
| Non-malignant polyp | 0.0102 | 0.595 | 0.9863 | -0.013 | 0.6435 | 0.9839 |
| Other | -0.0914 | 0.5913 | 0.8772 | 0.0965 | 0.6395 | 0.8801 |
| Operative approach |  |  |  |  |  |  |
| Minimally invasive (Reference) |  |  |  |  |  |  |
| Minimally invasive with open- or hand-assist | 0.2177 | 0.1058 | 0.0397 | -0.2355 | 0.1148 | 0.0402 |
| Minimally invasive with unplanned conversion to open | 0.9194 | 0.1278 | <0.001 | -0.9935 | 0.1432 | <0.001 |
| Open | 0.7937 | 0.1008 | <0.001 | -0.8597 | 0.1137 | <0.001 |
| Total operation time | 0.0014 | < 0.001 | 0.0002 | -0.0016 | < 0.001 | 0.0003 |
| Wound classification |  |  |  |  |  |  |
| Clean (Reference) |  |  |  |  |  |  |
| Clean/contaminated | -0.5583 | 0.2717 | 0.0399 | 0.609 | 0.2946 | 0.0387 |
| Contaminated | -0.4292 | 0.2942 | 0.1447 | 0.4691 | 0.3185 | 0.1408 |
| Dirty/infected | -0.1370 | 0.3195 | 0.6680 | 0.1517 | 0.3455 | 0.6606 |
| Any regional anesthesia use | 0.1191 | 0.089 | 0.1808 | -0.1288 | 0.0964 | 0.1815 |

The predictor "Operative approach" with the level "Other" (n = 71) was excluded because there were no events observed within this category. ASA PS, American Society of Anesthesiologists Physical Status; COPD, chronic obstructive pulmonary disease; GFR, glomerular filtration rate as calculated using Chronic Kidney Disease Epidemiology Collaboration (CKD-EPI) formula; IBD, Inflammatory bowel disease; IQR, interquartile range; SE, standard error.

| **S2.5 Table.** The Features and Coefficients for the Cox Proportional-Hazard Model for Pneumonia.   \|  \| **Cox PH** \| \| \| **AFT** \| \| \| \| --- \| --- \| --- \| --- \| --- \| --- \| --- \| \| **Feature** \| **Coefficient** \| **SE** \| **p-value** \| **Coefficient** \| **SE** \| **p-value** \| \| Intercept \|  \|  \|  \| 15.248 \| 1.0402 \| <0.001 \| \| Age \| 0.0302 \| 0.0027 \| <0.001 \| -0.0456 \| 0.0042 \| <0.001 \| \| Male sex \| 0.5587 \| 0.0559 \| <0.001 \| -0.8448 \| 0.087 \| <0.001 \| \| ASA classification \|  \|  \|  \|  \|  \|  \| \| I (Reference) \|  \|  \|  \|  \|  \|  \| \| II \| 0.2647 \| 0.3837 \| 0.4903 \| -0.3978 \| 0.5782 \| 0.4914 \| \| III \| 0.9436 \| 0.3828 \| 0.0137 \| -1.4214 \| 0.578 \| 0.0139 \| \| IV \| 1.3917 \| 0.3918 \| 0.0004 \| -2.0986 \| 0.5929 \| 0.0004 \| \| Body Mass Index \| -0.0129 \| 0.0045 \| 0.0040 \| 0.0195 \| 0.0068 \| 0.0040 \| \| Diabetes \|  \|  \|  \|  \|  \|  \| \| Insulin \| -0.1342 \| 0.1122 \| 0.2316 \| 0.2052 \| 0.1692 \| 0.2250 \| \| Non-Insulin \| -0.0267 \| 0.0803 \| 0.7397 \| 0.0414 \| 0.1211 \| 0.7324 \| \| History of severe COPD \| 0.7118 \| 0.0809 \| <0.001 \| -1.0783 \| 0.1251 \| <0.001 \| \| Smoker within one year preoperatively \| 0.6772 \| 0.0655 \| <0.001 \| -1.0212 \| 0.1024 \| <0.001 \| \| Congestive heart failure, within 30 days before surgery \| 0.2581 \| 0.183 \| 0.1585 \| -0.3846 \| 0.276 \| 0.1634 \| \| Preoperative renal failure (preoperative acute renal failure, dialysis, and/or GFR ≤ 60mL/min) \| 0.1713 \| 0.0668 \| 0.0104 \| -0.2578 \| 0.1009 \| 0.0106 \| \| Hypertension requiring medication \| 0.0444 \| 0.0616 \| 0.4718 \| -0.0672 \| 0.0929 \| 0.4696 \| \| Preoperative functional health status \|  \|  \|  \|  \|  \|  \| \| Independent (Reference) \|  \|  \|  \|  \|  \|  \| \| Partially dependent \| 0.7570 \| 0.1336 \| <0.001 \| -1.1465 \| 0.2035 \| <0.001 \| \| Totally dependent \| 2.1490 \| 0.2227 \| <0.001 \| -3.2658 \| 0.3461 \| <0.001 \| \| Dyspnea \|  \|  \|  \|  \|  \|  \| \| At rest \| 0.2927 \| 0.2871 \| 0.3079 \| -0.4338 \| 0.4328 \| 0.3162 \| \| Moderate exertion \| 0.3539 \| 0.0818 \| <0.001 \| -0.5327 \| 0.124 \| <0.001 \| \| Bleeding disorders or on anticoagulants \| 0.2303 \| 0.1341 \| 0.0859 \| -0.3488 \| 0.2022 \| 0.0846 \| \| Steroid/immunosuppressant use for a chronic condition \| 0.2776 \| 0.1013 \| 0.0061 \| -0.4223 \| 0.1531 \| 0.0058 \| \| Chemotherapy within 90 days preoperatively \| -0.3296 \| 0.1308 \| 0.0118 \| 0.498 \| 0.1975 \| 0.0117 \| \| Ascites within 30 days preoperatively \| 0.3018 \| 0.4111 \| 0.4629 \| -0.4483 \| 0.6195 \| 0.4693 \| \| Primary indication for colectomy \|  \|  \|  \|  \|  \|  \| \| Bleeding (Reference) \|  \|  \|  \|  \|  \|  \| \| Cancer \| -0.4384 \| 0.4117 \| 0.2870 \| 0.6661 \| 0.6206 \| 0.2831 \| \| Diverticulitis \| -0.8450 \| 0.42 \| 0.0442 \| 1.2803 \| 0.6337 \| 0.0434 \| \| IBD \| -0.3710 \| 0.4318 \| 0.3902 \| 0.567 \| 0.6507 \| 0.3836 \| \| Non-malignant polyp \| -0.2882 \| 0.4177 \| 0.4901 \| 0.4372 \| 0.6294 \| 0.4873 \| \| Other \| -0.3868 \| 0.4159 \| 0.3525 \| 0.5896 \| 0.6269 \| 0.3470 \| \| Operative approach \|  \|  \|  \|  \|  \|  \| \| Minimally invasive (Reference) \|  \|  \|  \|  \|  \|  \| \| Minimally invasive with open- or hand-assist \| 0.0771 \| 0.0721 \| 0.2849 \| -0.116 \| 0.1086 \| 0.2854 \| \| Minimally invasive with unplanned conversion to open \| 0.7102 \| 0.0902 \| <0.001 \| -1.0732 \| 0.1387 \| <0.001 \| \| Open \| 0.6347 \| 0.07 \| <0.001 \| -0.9601 \| 0.1085 \| <0.001 \| \| Other \| 1.1766 \| 0.7091 \| 0.0970 \| -1.778 \| 1.0693 \| 0.0964 \| \| Total operation time \| 0.0018 \| < 0.001 \| <0.001 \| -0.0028 \| < 0.001 \| <0.001 \| \| Wound classification \|  \|  \|  \|  \|  \|  \| \| Clean (Reference) \|  \|  \|  \|  \|  \|  \| \| Clean/contaminated \| 0.1528 \| 0.2799 \| 0.5851 \| -0.2258 \| 0.4217 \| 0.5924 \| \| Contaminated \| 0.3917 \| 0.2884 \| 0.1744 \| -0.5865 \| 0.4348 \| 0.1773 \| \| Dirty/infected \| 0.4230 \| 0.3008 \| 0.1596 \| -0.6342 \| 0.4535 \| 0.1620 \| \| Any regional anesthesia use \| 0.0435 \| 0.0634 \| 0.4925 \| -0.0664 \| 0.0955 \| 0.4868 \|   ASA PS, American Society of Anesthesiologists Physical Status; COPD, chronic obstructive pulmonary disease; GFR, glomerular filtration rate as calculated using Chronic Kidney Disease Epidemiology Collaboration (CKD-EPI) formula; IBD, Inflammatory bowel disease; IQR, interquartile range; SE, standard error. |
| --- | --- | --- | --- | --- | --- | --- | --- | --- | --- | --- | --- | --- | --- | --- | --- | --- | --- | --- | --- | --- | --- | --- | --- | --- | --- | --- | --- | --- | --- | --- | --- | --- | --- | --- | --- | --- | --- | --- | --- | --- | --- | --- | --- | --- | --- | --- | --- | --- | --- | --- | --- | --- | --- | --- | --- | --- | --- | --- | --- | --- | --- | --- | --- | --- | --- | --- | --- | --- | --- | --- | --- | --- | --- | --- | --- | --- | --- | --- | --- | --- | --- | --- | --- | --- | --- | --- | --- | --- | --- | --- | --- | --- | --- | --- | --- | --- | --- | --- | --- | --- | --- | --- | --- | --- | --- | --- | --- | --- | --- | --- | --- | --- | --- | --- | --- | --- | --- | --- | --- | --- | --- | --- | --- | --- | --- | --- | --- | --- | --- | --- | --- | --- | --- | --- | --- | --- | --- | --- | --- | --- | --- | --- | --- | --- | --- | --- | --- | --- | --- | --- | --- | --- | --- | --- | --- | --- | --- | --- | --- | --- | --- | --- | --- | --- | --- | --- | --- | --- | --- | --- | --- | --- | --- | --- | --- | --- | --- | --- | --- | --- | --- | --- | --- | --- | --- | --- | --- | --- | --- | --- | --- | --- | --- | --- | --- | --- | --- | --- | --- | --- | --- | --- | --- | --- | --- | --- | --- | --- | --- | --- | --- | --- | --- | --- | --- | --- | --- | --- | --- | --- | --- | --- | --- | --- | --- | --- | --- | --- | --- | --- | --- | --- | --- | --- | --- | --- | --- | --- | --- | --- | --- | --- | --- | --- | --- | --- | --- | --- | --- | --- | --- | --- | --- | --- | --- | --- | --- | --- | --- | --- | --- | --- | --- | --- | --- | --- | --- | --- | --- | --- | --- | --- | --- | --- | --- | --- | --- | --- | --- | --- | --- | --- | --- | --- | --- | --- | --- | --- | --- | --- | --- | --- | --- | --- | --- | --- | --- | --- | --- | --- | --- | --- | --- | --- | --- | --- | --- | --- | --- | --- | --- | --- | --- | --- | --- | --- | --- | --- | --- | --- | --- | --- | --- | --- | --- | --- | --- | --- | --- | --- | --- | --- | --- | --- | --- | --- | --- | --- | --- | --- | --- | --- | --- | --- | --- | --- | --- | --- | --- | --- |

**S2.6 Table.** The Features and Coefficients for the Cox Proportional-Hazard Model for Cerebrovascular Event.

|  | **Cox PH** | | | **AFT** | | |
| --- | --- | --- | --- | --- | --- | --- |
| **Feature** | **Coefficient** | **SE** | **p-value** | **Coefficient** | **SE** | **p-value** |
| Intercept |  |  |  | 15.5942 | 1.553 | <0.001 |
| Age | 0.0542 | 0.0078 | <0.001 | -0.0596 | 0.0086 | <0.001 |
| Male sex | 0.0504 | 0.1392 | 0.7173 | -0.0556 | 0.1532 | 0.7165 |
| ASA classification |  |  |  |  |  |  |
| II (Reference) |  |  |  |  |  |  |
| III | 0.5530 | 0.1922 | 0.0040 | -0.6083 | 0.2114 | 0.0040 |
| IV | 0.7423 | 0.2837 | 0.0089 | -0.8166 | 0.3121 | 0.0089 |
| Body Mass Index | -0.0021 | 0.0113 | 0.8561 | 0.0023 | 0.0125 | 0.8564 |
| Diabetes |  |  |  |  |  |  |
| Insulin | 0.2222 | 0.2399 | 0.3544 | -0.2442 | 0.264 | 0.3550 |
| Non-Insulin | 0.1438 | 0.1845 | 0.4356 | -0.1581 | 0.2029 | 0.4360 |
| History of severe COPD | 0.0282 | 0.2469 | 0.9092 | -0.0297 | 0.2717 | 0.9130 |
| Smoker within one year preoperatively | 0.1630 | 0.2203 | 0.4593 | -0.1794 | 0.2423 | 0.4592 |
| Congestive heart failure, within 30 days before surgery | 0.7133 | 0.3597 | 0.0474 | -0.7857 | 0.3958 | 0.0472 |
| Preoperative renal failure (preoperative acute renal failure, dialysis, and/or GFR ≤ 60mL/min) | 0.3498 | 0.1537 | 0.0229 | -0.3855 | 0.1691 | 0.0226 |
| Hypertension requiring medication | 0.5197 | 0.1775 | 0.0034 | -0.5719 | 0.1952 | 0.0034 |
| Preoperative functional health status |  |  |  |  |  |  |
| Independent (Reference) |  |  |  |  |  |  |
| Partially dependent | 1.0300 | 0.2696 | 0.0001 | -1.1355 | 0.2966 | 0.0001 |
| Totally dependent | 1.8895 | 0.5896 | 0.0014 | -2.0861 | 0.6488 | 0.0013 |
| Dyspnea |  |  |  |  |  |  |
| At rest | 0.4891 | 0.7303 | 0.5031 | -0.5397 | 0.8034 | 0.5018 |
| Moderate exertion | 0.4648 | 0.1955 | 0.0174 | -0.5124 | 0.2151 | 0.0172 |
| Bleeding disorders or on anticoagulants | -0.1439 | 0.3675 | 0.6954 | 0.16 | 0.4044 | 0.6924 |
| Steroid/immunosuppressant use for a chronic condition | 0.1498 | 0.2863 | 0.6009 | -0.1651 | 0.3149 | 0.6000 |
| Chemotherapy within 90 days preoperatively | -0.7290 | 0.4611 | 0.1139 | 0.8023 | 0.5072 | 0.1137 |
| Primary indication for colectomy |  |  |  |  |  |  |
| Bleeding (Reference) |  |  |  |  |  |  |
| Cancer | -1.0132 | 0.7197 | 0.1592 | 1.1229 | 0.7918 | 0.1562 |
| Diverticulitis | -1.2665 | 0.7505 | 0.0915 | 1.402 | 0.8257 | 0.0895 |
| IBD | -0.9116 | 0.8347 | 0.2748 | 1.0115 | 0.9183 | 0.2707 |
| Non-malignant polyp | -1.1245 | 0.7474 | 0.1324 | 1.2452 | 0.8222 | 0.1299 |
| Other | -1.2474 | 0.7466 | 0.0947 | 1.3812 | 0.8213 | 0.0926 |
| Operative approach |  |  |  |  |  |  |
| Minimally invasive (Reference) |  |  |  |  |  |  |
| Minimally invasive with open- or hand-assist | 0.0792 | 0.1714 | 0.6438 | -0.0874 | 0.1885 | 0.6431 |
| Minimally invasive with unplanned conversion to open | 0.5116 | 0.234 | 0.0288 | -0.5627 | 0.2574 | 0.0288 |
| Open | 0.2999 | 0.1837 | 0.1025 | -0.3302 | 0.202 | 0.1022 |
| Total operation time | 0.0008 | < 0.001 | 0.3012 | -0.0009 | < 0.001 | 0.3004 |
| Wound classification |  |  |  |  |  |  |
| Clean (Reference) |  |  |  |  |  |  |
| Clean/contaminated | 0.9243 | 1.0035 | 0.3570 | -1.0177 | 1.1039 | 0.3566 |
| Contaminated | 0.8708 | 1.0268 | 0.3964 | -0.9616 | 1.1295 | 0.3946 |
| Dirty/infected | 0.8577 | 1.0665 | 0.4212 | -0.9443 | 1.1731 | 0.4209 |
| Any regional anesthesia use | -0.2336 | 0.178 | 0.1895 | 0.2574 | 0.1958 | 0.1886 |

The predictor "Operative approach" with the level "Other", “ASA classification” with the level “I”, and “Ascites within 30 days preoperatively” (n =2734) were excluded because there were no events observed within this category. ASA PS, American Society of Anesthesiologists Physical Status; COPD, chronic obstructive pulmonary disease; GFR, glomerular filtration rate as calculated using Chronic Kidney Disease Epidemiology Collaboration (CKD-EPI) formula; IBD, Inflammatory bowel disease; IQR, interquartile range; SE, standard error.

| **S2.7 Table.** The Features and Coefficients for the Cox Proportional-Hazard Model for Venous Thromboembolism   \|  \| **Cox PH** \| \| \| **AFT** \| \| \| \| --- \| --- \| --- \| --- \| --- \| --- \| --- \| \| **Feature** \| **Coefficient** \| **SE** \| **p-value** \| **Coefficient** \| **SE** \| **p-value** \| \| Intercept \|  \|  \|  \| 10.0124 \| 0.6535 \| <0.001 \| \| Age \| 0.0091 \| 0.0023 \| <0.001 \| -0.0092 \| 0.0024 \| 0.0001 \| \| Male sex \| 0.0527 \| 0.0527 \| 0.3166 \| -0.0538 \| 0.0536 \| 0.3160 \| \| ASA classification \|  \|  \|  \|  \|  \|  \| \| I (Reference) \|  \|  \|  \|  \|  \|  \| \| II \| 0.2507 \| 0.2549 \| 0.3253 \| -0.2553 \| 0.2595 \| 0.3252 \| \| III \| 0.5425 \| 0.2563 \| 0.0343 \| -0.5525 \| 0.2613 \| 0.0345 \| \| IV \| 0.5505 \| 0.2862 \| 0.0544 \| -0.5604 \| 0.2917 \| 0.0547 \| \| Body Mass Index \| 0.0238 \| 0.0038 \| <0.001 \| -0.0243 \| 0.0039 \| <0.001 \| \| Diabetes \|  \|  \|  \|  \|  \|  \| \| Insulin \| -0.1869 \| 0.1234 \| 0.1298 \| 0.1903 \| 0.1257 \| 0.1301 \| \| Non-Insulin \| -0.0513 \| 0.0843 \| 0.5431 \| 0.0524 \| 0.0859 \| 0.5420 \| \| History of severe COPD \| 0.1684 \| 0.1203 \| 0.1615 \| -0.1715 \| 0.1225 \| 0.1614 \| \| Smoker within one year preoperatively \| -0.0453 \| 0.0749 \| 0.5450 \| 0.0461 \| 0.0762 \| 0.5452 \| \| Congestive heart failure, within 30 days before surgery \| 0.4457 \| 0.2584 \| 0.0845 \| -0.4548 \| 0.2633 \| 0.0841 \| \| Preoperative renal failure (preoperative acute renal failure, dialysis, and/or GFR ≤ 60mL/min) \| -0.1128 \| 0.0793 \| 0.1551 \| 0.1148 \| 0.0808 \| 0.1554 \| \| Hypertension requiring medication \| -0.1279 \| 0.0597 \| 0.0323 \| 0.1305 \| 0.0609 \| 0.0322 \| \| Preoperative functional health status \|  \|  \|  \|  \|  \|  \| \| Independent (Reference) \|  \|  \|  \|  \|  \|  \| \| Partially dependent \| 0.5015 \| 0.1848 \| 0.0066 \| -0.5107 \| 0.1885 \| 0.0068 \| \| Totally dependent \| 0.6656 \| 0.5023 \| 0.1851 \| -0.6785 \| 0.5116 \| 0.1847 \| \| Dyspnea \|  \|  \|  \|  \|  \|  \| \| At rest \| -0.2423 \| 0.5083 \| 0.6336 \| 0.2474 \| 0.5175 \| 0.6326 \| \| Moderate exertion \| -0.0768 \| 0.1101 \| 0.4855 \| 0.0783 \| 0.1121 \| 0.4846 \| \| Bleeding disorders or on anticoagulants \| 0.3832 \| 0.1458 \| 0.0086 \| -0.3906 \| 0.1487 \| 0.0086 \| \| Steroid/immunosuppressant use for a chronic condition \| 0.337 \| 0.0974 \| 0.0005 \| -0.3435 \| 0.0996 \| 0.0006 \| \| Chemotherapy within 90 days preoperatively \| -0.2422 \| 0.1184 \| 0.0408 \| 0.2464 \| 0.1207 \| 0.0412 \| \| Ascites within 30 days preoperatively \| -0.4547 \| 0.7086 \| 0.5211 \| 0.4614 \| 0.7214 \| 0.5224 \| \| Primary indication for colectomy \|  \|  \|  \|  \|  \|  \| \| Bleeding (Reference) \|  \|  \|  \|  \|  \|  \| \| Cancer \| -0.6334 \| 0.4498 \| 0.1590 \| 0.6456 \| 0.4581 \| 0.1588 \| \| Diverticulitis \| -0.9156 \| 0.454 \| 0.0437 \| 0.9328 \| 0.4628 \| 0.0438 \| \| IBD \| -0.2808 \| 0.461 \| 0.5425 \| 0.2864 \| 0.4693 \| 0.5418 \| \| Non-malignant polyp \| -0.6733 \| 0.4559 \| 0.1398 \| 0.6861 \| 0.4645 \| 0.1396 \| \| Other \| -0.624 \| 0.4534 \| 0.1687 \| 0.6357 \| 0.4618 \| 0.1687 \| \| Operative approach \|  \|  \|  \|  \|  \|  \| \| Minimally invasive (Reference) \|  \|  \|  \|  \|  \|  \| \| Minimally invasive with open- or hand-assist \| 0.186 \| 0.066 \| 0.0049 \| -0.1894 \| 0.0674 \| 0.0050 \| \| Minimally invasive with unplanned conversion to open \| 0.6409 \| 0.0876 \| <0.001 \| -0.6528 \| 0.0907 \| <0.001 \| \| Open \| 0.4964 \| 0.0711 \| <0.001 \| -0.5055 \| 0.0735 \| <0.001 \| \| Other \| 0.3499 \| 1.0012 \| 0.7268 \| -0.3561 \| 1.0192 \| 0.7268 \| \| Total operation time \| 0.0026 \| < 0.001 \| <0.001 \| -0.0026 \| < 0.001 \| <0.001 \| \| Wound classification \|  \|  \|  \|  \|  \|  \| \| Clean (Reference) \|  \|  \|  \|  \|  \|  \| \| Clean/contaminated \| 0.2099 \| 0.3031 \| 0.4886 \| -0.2142 \| 0.3086 \| 0.4876 \| \| Contaminated \| 0.309 \| 0.3107 \| 0.3199 \| -0.3149 \| 0.3164 \| 0.3196 \| \| Dirty/infected \| 0.548 \| 0.318 \| 0.0848 \| -0.5585 \| 0.324 \| 0.0848 \| \| Any regional anesthesia use \| 0.0224 \| 0.062 \| 0.7175 \| -0.0228 \| 0.0631 \| 0.7175 \| |
| --- | --- | --- | --- | --- | --- | --- | --- | --- | --- | --- | --- | --- | --- | --- | --- | --- | --- | --- | --- | --- | --- | --- | --- | --- | --- | --- | --- | --- | --- | --- | --- | --- | --- | --- | --- | --- | --- | --- | --- | --- | --- | --- | --- | --- | --- | --- | --- | --- | --- | --- | --- | --- | --- | --- | --- | --- | --- | --- | --- | --- | --- | --- | --- | --- | --- | --- | --- | --- | --- | --- | --- | --- | --- | --- | --- | --- | --- | --- | --- | --- | --- | --- | --- | --- | --- | --- | --- | --- | --- | --- | --- | --- | --- | --- | --- | --- | --- | --- | --- | --- | --- | --- | --- | --- | --- | --- | --- | --- | --- | --- | --- | --- | --- | --- | --- | --- | --- | --- | --- | --- | --- | --- | --- | --- | --- | --- | --- | --- | --- | --- | --- | --- | --- | --- | --- | --- | --- | --- | --- | --- | --- | --- | --- | --- | --- | --- | --- | --- | --- | --- | --- | --- | --- | --- | --- | --- | --- | --- | --- | --- | --- | --- | --- | --- | --- | --- | --- | --- | --- | --- | --- | --- | --- | --- | --- | --- | --- | --- | --- | --- | --- | --- | --- | --- | --- | --- | --- | --- | --- | --- | --- | --- | --- | --- | --- | --- | --- | --- | --- | --- | --- | --- | --- | --- | --- | --- | --- | --- | --- | --- | --- | --- | --- | --- | --- | --- | --- | --- | --- | --- | --- | --- | --- | --- | --- | --- | --- | --- | --- | --- | --- | --- | --- | --- | --- | --- | --- | --- | --- | --- | --- | --- | --- | --- | --- | --- | --- | --- | --- | --- | --- | --- | --- | --- | --- | --- | --- | --- | --- | --- | --- | --- | --- | --- | --- | --- | --- | --- | --- | --- | --- | --- | --- | --- | --- | --- | --- | --- | --- | --- | --- | --- | --- | --- | --- | --- | --- | --- | --- | --- | --- | --- | --- | --- | --- | --- | --- | --- | --- | --- | --- | --- | --- | --- | --- | --- | --- | --- | --- | --- | --- | --- | --- | --- | --- | --- | --- | --- | --- | --- | --- | --- | --- | --- | --- | --- | --- | --- | --- | --- | --- | --- | --- | --- | --- | --- | --- | --- | --- | --- | --- | --- | --- | --- | --- | --- | --- | --- | --- | --- |

ASA PS, American Society of Anesthesiologists Physical Status; COPD, chronic obstructive pulmonary disease; GFR, glomerular filtration rate as calculated using Chronic Kidney Disease Epidemiology Collaboration (CKD-EPI) formula; IBD, Inflammatory bowel disease; IQR, interquartile range; SE, standard error.

**S2.8 Table.** The Features and Coefficients for the Cox Proportional-Hazard Model for Acute Renal Failure.

|  | **Cox PH** | | | **AFT** | | |
| --- | --- | --- | --- | --- | --- | --- |
| **Feature** | **Coefficient** | **SE** | **p-value** | **Coefficient** | **SE** | **p-value** |
| Intercept |  |  |  | 16.2577 | 1.9974 | <0.001 |
| Age | 0.0223 | 0.0056 | <0.001 | -0.0271 | 0.007 | 0.0001 |
| Male sex | 0.7109 | 0.1172 | <0.001 | -0.8635 | 0.1497 | <0.001 |
| ASA classification |  |  |  |  |  |  |
| I (Reference) |  |  |  |  |  |  |
| II | 0.364 | 1.0114 | 0.7189 | -0.442 | 1.2283 | 0.7190 |
| III | 1.047 | 1.009 | 0.2995 | -1.2714 | 1.2271 | 0.3002 |
| IV | 1.4205 | 1.0224 | 0.1647 | -1.7258 | 1.2449 | 0.1656 |
| Body Mass Index | 0.0229 | 0.008 | 0.0042 | -0.0278 | 0.0098 | 0.0047 |
| Diabetes |  |  |  |  |  |  |
| Insulin | 0.8907 | 0.151 | <0.001 | -1.0823 | 0.1923 | <0.001 |
| Non-Insulin | 0.2664 | 0.1479 | 0.0717 | -0.3238 | 0.1805 | 0.0728 |
| History of severe COPD | -0.1676 | 0.212 | 0.4293 | 0.2039 | 0.2577 | 0.4289 |
| Smoker within one year preoperatively | 0.6315 | 0.1396 | <0.001 | -0.7672 | 0.1744 | <0.001 |
| Congestive heart failure, within 30 days before surgery | 0.8565 | 0.2806 | 0.0023 | -1.039 | 0.3453 | 0.0026 |
| Preoperative renal failure (preoperative acute renal failure, dialysis, and/or GFR ≤ 60mL/min) | 0.5387 | 0.1258 | <0.001 | -0.654 | 0.1567 | <0.001 |
| Hypertension requiring medication | 0.5217 | 0.141 | 0.0002 | -0.6335 | 0.1746 | 0.0003 |
| Preoperative functional health status |  |  |  |  |  |  |
| Independent (Reference) |  |  |  |  |  |  |
| Partially dependent | 0.298 | 0.3135 | 0.3417 | -0.3629 | 0.3811 | 0.3410 |
| Totally dependent | 0.2945 | 1.0055 | 0.7696 | -0.3582 | 1.2212 | 0.7693 |
| Dyspnea |  |  |  |  |  |  |
| At rest | 0.687 | 0.5275 | 0.1928 | -0.8368 | 0.642 | 0.1924 |
| Moderate exertion | 0.1502 | 0.1739 | 0.3876 | -0.1828 | 0.2114 | 0.3870 |
| Bleeding disorders or on anticoagulants | 0.4815 | 0.2289 | 0.0354 | -0.5844 | 0.2797 | 0.0367 |
| Steroid/immunosuppressant use for a chronic condition | -0.1036 | 0.2344 | 0.6585 | 0.1265 | 0.2848 | 0.6569 |
| Chemotherapy within 90 days preoperatively | 0.4484 | 0.1939 | 0.0207 | -0.5444 | 0.2372 | 0.0217 |
| Ascites within 30 days preoperatively | 1.0615 | 0.5872 | 0.0706 | -1.2926 | 0.7163 | 0.0712 |
| Primary indication for colectomy |  |  |  |  |  |  |
| Bleeding (Reference) |  |  |  |  |  |  |
| Cancer | 0.0967 | 1.0074 | 0.9236 | -0.117 | 1.2233 | 0.9238 |
| Diverticulitis | -0.4956 | 1.0265 | 0.6293 | 0.6021 | 1.2469 | 0.6292 |
| IBD | 0.4502 | 1.0458 | 0.6668 | -0.5469 | 1.2702 | 0.6668 |
| Non-malignant polyp | 0.4015 | 1.0154 | 0.6926 | -0.4874 | 1.2333 | 0.6927 |
| Other | -0.1513 | 1.0186 | 0.8819 | 0.1844 | 1.2369 | 0.8815 |
| Operative approach |  |  |  |  |  |  |
| Minimally invasive (Reference) |  |  |  |  |  |  |
| Minimally invasive with open- or hand-assist | 0.1049 | 0.144 | 0.4664 | -0.1274 | 0.175 | 0.4664 |
| Minimally invasive with unplanned conversion to open | 0.4913 | 0.1897 | 0.0096 | -0.5964 | 0.2326 | 0.0104 |
| Open | 0.7155 | 0.1398 | <0.001 | -0.8693 | 0.176 | <0.001 |
| Total operation time | 0.0023 | < 0.001 | <0.001 | -0.0028 | < 0.001 | <0.001 |
| Wound classification |  |  |  |  |  |  |
| Clean (Reference) |  |  |  |  |  |  |
| Clean/contaminated | -0.3616 | 0.4519 | 0.4236 | 0.4392 | 0.5492 | 0.4239 |
| Contaminated | -0.1452 | 0.4754 | 0.7600 | 0.1768 | 0.5773 | 0.7595 |
| Dirty/infected | -0.0844 | 0.5151 | 0.8698 | 0.1021 | 0.6255 | 0.8704 |
| Any regional anesthesia use | -0.0332 | 0.1304 | 0.7993 | 0.0401 | 0.1584 | 0.8004 |

The predictor "Operative approach" with the level "Other" (n = 71) was excluded because there were no events observed within this category. ASA PS, American Society of Anesthesiologists Physical Status; COPD, chronic obstructive pulmonary disease; GFR, glomerular filtration rate as calculated using Chronic Kidney Disease Epidemiology Collaboration (CKD-EPI) formula; IBD, Inflammatory bowel disease; IQR, interquartile range; SE, standard error.

**S2.9 Table.** The Features and Coefficients for the Cox Proportional-Hazard Model for Sepsis or Septic Shock.

|  | **Cox PH** | | | **AFT** | | |
| --- | --- | --- | --- | --- | --- | --- |
| **Feature** | **Coefficient** | **SE** | **p-value** | **Coefficient** | **SE** | **p-value** |
| Intercept |  |  |  | 9.3832 | 0.5126 | <0.001 |
| Age | -0.0001 | 0.0016 | 0.9711 | < 0.001 | 0.002 | 0.9627 |
| Male sex | 0.2416 | 0.0355 | <0.001 | -0.3075 | 0.0454 | <0.001 |
| ASA classification |  |  |  |  |  |  |
| I (Reference) |  |  |  |  |  |  |
| II | 0.2028 | 0.175 | 0.2465 | -0.257 | 0.2224 | 0.2479 |
| III | 0.5196 | 0.1759 | 0.0031 | -0.6607 | 0.2238 | 0.0032 |
| IV | 0.8805 | 0.1892 | <0.001 | -1.1221 | 0.2413 | <0.001 |
| Body Mass Index | < 0.001 | 0.0027 | 0.8426 | -7.00E-04 | 0.0034 | 0.8379 |
| Diabetes |  |  |  |  |  |  |
| Insulin | 0.0500 | 0.0742 | 0.5004 | -0.066 | 0.0943 | 0.4844 |
| Non-Insulin | 0.044 | 0.0552 | 0.4252 | -0.0572 | 0.0701 | 0.4147 |
| History of severe COPD | 0.2473 | 0.0718 | 0.0006 | -0.3147 | 0.0914 | 0.0006 |
| Smoker within one year preoperatively | 0.3284 | 0.044 | <0.001 | -0.4183 | 0.0564 | <0.001 |
| Congestive heart failure, within 30 days before surgery | 0.2329 | 0.162 | 0.1505 | -0.2988 | 0.206 | 0.1470 |
| Preoperative renal failure (preoperative acute renal failure, dialysis, and/or GFR ≤ 60mL/min) | 0.1124 | 0.0499 | 0.0242 | -0.1431 | 0.0634 | 0.0241 |
| Hypertension requiring medication | 0.1238 | 0.0406 | 0.0023 | -0.158 | 0.0517 | 0.0022 |
| Preoperative functional health status |  |  |  |  |  |  |
| Independent (Reference) |  |  |  |  |  |  |
| Partially dependent | 0.5103 | 0.1164 | <0.001 | -0.6487 | 0.1484 | <0.001 |
| Totally dependent | 1.0405 | 0.2692 | 0.0001 | -1.3301 | 0.3429 | 0.0001 |
| Dyspnea |  |  |  |  |  |  |
| At rest | 0.0509 | 0.2738 | 0.8524 | -0.0634 | 0.3481 | 0.8556 |
| Moderate exertion | 0.2144 | 0.0651 | 0.0010 | -0.2748 | 0.0829 | 0.0009 |
| Bleeding disorders or on anticoagulants | 0.2999 | 0.0964 | 0.0019 | -0.3852 | 0.1226 | 0.0017 |
| Steroid/immunosuppressant use for a chronic condition | 0.2820 | 0.0652 | <0.001 | -0.3585 | 0.0831 | <0.001 |
| Chemotherapy within 90 days preoperatively | 0.0302 | 0.0728 | 0.6783 | -0.0383 | 0.0925 | 0.6793 |
| Ascites within 30 days preoperatively | 0.9902 | 0.2204 | <0.001 | -1.2715 | 0.2810 | <0.001 |
| Primary indication for colectomy |  |  |  |  |  |  |
| Bleeding (Reference) |  |  |  |  |  |  |
| Cancer | -0.7928 | 0.2695 | 0.0033 | 1.013 | 0.3431 | 0.0032 |
| Diverticulitis | -0.9691 | 0.2725 | 0.0004 | 1.2383 | 0.3471 | 0.0004 |
| IBD | -0.6796 | 0.2789 | 0.0148 | 0.8692 | 0.3548 | 0.0143 |
| Non-malignant polyp | -0.7300 | 0.2741 | 0.0077 | 0.9333 | 0.3488 | 0.0075 |
| Other | -0.6746 | 0.2717 | 0.0130 | 0.8632 | 0.3457 | 0.0125 |
| Operative approach |  |  |  |  |  |  |
| Minimally invasive (Reference) |  |  |  |  |  |  |
| Minimally invasive with open- or hand-assist | 0.2592 | 0.0457 | <0.001 | -0.3302 | 0.0584 | <0.001 |
| Minimally invasive with unplanned conversion to open | 0.6610 | 0.0606 | <0.001 | -0.8421 | 0.0784 | <0.001 |
| Open | 0.6907 | 0.0469 | <0.001 | -0.8812 | 0.0615 | <0.001 |
| Other | 0.7731 | 0.5784 | 0.1814 | -0.9794 | 0.7354 | 0.1829 |
| Total operation time | 0.0022 | < 0.001 | <0.001 | -0.0028 | < 0.001 | <0.001 |
| Wound classification |  |  |  |  |  |  |
| Clean (Reference) |  |  |  |  |  |  |
| Clean/contaminated | 0.1936 | 0.2053 | 0.3458 | -0.2449 | 0.261 | 0.3481 |
| Contaminated | 0.5728 | 0.2093 | 0.0062 | -0.7282 | 0.2663 | 0.0063 |
| Dirty/infected | 0.9353 | 0.2124 | <0.001 | -1.1872 | 0.2708 | <0.001 |
| Any regional anesthesia use | 0.0013 | 0.0416 | 0.9752 | -9.00E-04 | 0.0529 | 0.9861 |

ASA PS, American Society of Anesthesiologists Physical Status; COPD, chronic obstructive pulmonary disease; GFR, glomerular filtration rate as calculated using Chronic Kidney Disease Epidemiology Collaboration (CKD-EPI) formula; IBD, Inflammatory bowel disease; IQR, interquartile range; SE, standard error.

**S2.10 Table.** The Features and Coefficients for the Cox Proportional-Hazard Model for Myocardial Infarction.

|  | **Cox PH** | | | **AFT** | | |
| --- | --- | --- | --- | --- | --- | --- |
| **Feature** | **Coefficient** | **SE** | **p-value** | **Coefficient** | **SE** | **p-value** |
| Intercept |  |  |  | 23.2268 | 2.1924 | <0.001 |
| Age | 0.0479 | 0.0045 | <0.001 | -0.0925 | 0.0094 | <0.001 |
| Male sex | 0.4257 | 0.0828 | <0.001 | -0.8226 | 0.1629 | <0.001 |
| ASA classification |  |  |  |  |  |  |
| I (Reference) |  |  |  |  |  |  |
| II | 0.2439 | 0.716 | 0.7333 | -0.4704 | 1.3817 | 0.7335 |
| III | 0.8814 | 0.7142 | 0.2172 | -1.7008 | 1.3798 | 0.2177 |
| IV | 1.8231 | 0.7211 | 0.0115 | -3.5200 | 1.3984 | 0.0118 |
| Body Mass Index | -0.0061 | 0.0067 | 0.3620 | 0.0119 | 0.0129 | 0.3577 |
| Diabetes |  |  |  |  |  |  |
| Insulin | 0.2576 | 0.1366 | 0.0593 | -0.4969 | 0.2643 | 0.0601 |
| Non-Insulin | 0.0581 | 0.1103 | 0.5987 | -0.1122 | 0.2129 | 0.5982 |
| History of severe COPD | 0.1571 | 0.1359 | 0.2476 | -0.3058 | 0.2625 | 0.2441 |
| Smoker within one year preoperatively | 0.3658 | 0.1155 | 0.0015 | -0.7065 | 0.2246 | 0.0017 |
| Congestive heart failure, within 30 days before surgery | -0.0976 | 0.2777 | 0.7252 | 0.1892 | 0.5359 | 0.7240 |
| Preoperative renal failure (preoperative acute renal failure, dialysis, and/or GFR ≤ 60mL/min) | 0.3189 | 0.0909 | 0.0005 | -0.6165 | 0.1772 | 0.0005 |
| Hypertension requiring medication | 0.4803 | 0.102 | <0.001 | -0.9272 | 0.2002 | <0.001 |
| Preoperative functional health status |  |  |  |  |  |  |
| Independent (Reference) |  |  |  |  |  |  |
| Partially dependent | 0.1504 | 0.2213 | 0.4967 | -0.2897 | 0.4272 | 0.4978 |
| Totally dependent | 0.7582 | 0.5804 | 0.1914 | -1.4852 | 1.1215 | 0.1854 |
| Dyspnea |  |  |  |  |  |  |
| At rest | 0.4774 | 0.4218 | 0.2578 | -0.9216 | 0.8148 | 0.2580 |
| Moderate exertion | 0.3358 | 0.1181 | 0.0045 | -0.6484 | 0.2293 | 0.0047 |
| Bleeding disorders or on anticoagulants | 0.5494 | 0.164 | 0.0008 | -1.0628 | 0.3192 | 0.0009 |
| Steroid/immunosuppressant use for a chronic condition | 0.0640 | 0.1688 | 0.7045 | -0.1226 | 0.3257 | 0.7066 |
| Chemotherapy within 90 days preoperatively | -0.2392 | 0.1971 | 0.2250 | 0.4622 | 0.3808 | 0.2249 |
| Ascites within 30 days preoperatively | -0.0769 | 0.7112 | 0.9139 | 0.1600 | 1.3724 | 0.9072 |
| Primary indication for colectomy |  |  |  |  |  |  |
| Bleeding (Reference) |  |  |  |  |  |  |
| Cancer | -0.2611 | 0.5821 | 0.6537 | 0.5154 | 1.1235 | 0.6464 |
| Diverticulitis | -0.5067 | 0.5945 | 0.3940 | 0.9892 | 1.1478 | 0.3888 |
| IBD | -0.4573 | 0.6374 | 0.4731 | 0.8916 | 1.2303 | 0.4687 |
| Non-malignant polyp | -0.2976 | 0.5933 | 0.6160 | 0.5857 | 1.1451 | 0.6090 |
| Other | -0.6205 | 0.5942 | 0.2964 | 1.2095 | 1.1475 | 0.2919 |
| Operative approach |  |  |  |  |  |  |
| Minimally invasive (Reference) |  |  |  |  |  |  |
| Minimally invasive with open- or hand-assist | 0.0641 | 0.1037 | 0.5369 | -0.1246 | 0.2002 | 0.5338 |
| Minimally invasive with unplanned conversion to open | 0.4506 | 0.1408 | 0.0014 | -0.8719 | 0.2738 | 0.0015 |
| Open | 0.5197 | 0.1039 | <0.001 | -1.0048 | 0.2044 | <0.001 |
| Other | 1.0782 | 1.0031 | 0.2824 | -2.0807 | 1.9373 | 0.2828 |
| Total operation time | 0.0018 | < 0.001 | <0.001 | -0.0035 | < 0.001 | <0.001 |
| Wound classification |  |  |  |  |  |  |
| Clean (Reference) |  |  |  |  |  |  |
| Clean/contaminated | 0.1334 | 0.4113 | 0.7456 | -0.2571 | 0.7937 | 0.7460 |
| Contaminated | 0.3994 | 0.4255 | 0.3478 | -0.7697 | 0.8215 | 0.3488 |
| Dirty/infected | 0.3621 | 0.4519 | 0.4230 | -0.6976 | 0.8723 | 0.4239 |
| Any regional anesthesia use | 0.0405 | 0.0956 | 0.6716 | -0.0787 | 0.1846 | 0.6696 |

ASA PS, American Society of Anesthesiologists Physical Status; COPD, chronic obstructive pulmonary disease; GFR, glomerular filtration rate as calculated using Chronic Kidney Disease Epidemiology Collaboration (CKD-EPI) formula; IBD, Inflammatory bowel disease; IQR, interquartile range; SE, standard error.

**S2.11 Table.** Baseline Hazard of Cox Proportional-Hazard Models, Incorporating the Intercept.

| Day(s) after surgery | Readmission | Mortality | MI | Pneumonia | CVA | VTE | ARF | Sepsis |
| --- | --- | --- | --- | --- | --- | --- | --- | --- |
| T = 0 | 4.45e-05 | 7.00e-07 | 2.70e-06 | 2.20e-06 | 1.00e-06 | 3.11e-06 | 5.00e-07 | 1.61e-04 |
| T = 1 | 1.02e-04 | 1.70e-06 | 9.10e-06 | 2.12e-05 | 2.80e-06 | 5.60e-05 | 1.60e-06 | 5.20e-04 |
| T = 2 | 4.45e-04 | 3.00e-06 | 1.53e-05 | 6.93e-05 | 4.20e-06 | 1.17e-04 | 3.50e-06 | 9.90e-04 |
| T = 3 | 1.71e-03 | 5.10e-06 | 1.93e-05 | 1.24e-04 | 5.20e-06 | 1.91e-04 | 5.30e-06 | 1.59e-03 |
| T = 4 | 4.35e-03 | 7.30e-06 | 2.22e-05 | 1.66e-04 | 6.40e-06 | 2.61e-04 | 6.40e-06 | 2.20e-03 |
| T = 5 | 8.46e-03 | 9.10e-06 | 2.40e-05 | 1.94e-04 | 6.80e-06 | 3.36e-04 | 7.80e-06 | 2.86e-03 |
| T = 6 | 1.28e-02 | 1.12e-05 | 2.55e-05 | 2.23e-04 | 7.60e-06 | 4.08e-04 | 9.30e-06 | 3.50e-03 |
| T = 7 | 1.70e-02 | 1.34e-05 | 2.68e-05 | 2.48e-04 | 8.20e-06 | 4.99e-04 | 1.06e-05 | 4.26e-03 |
| T = 8 | 2.15e-02 | 1.58e-05 | 2.74e-05 | 2.65e-04 | 9.30e-06 | 5.79e-04 | 1.23e-05 | 4.86e-03 |
| T = 9 | 2.58e-02 | 1.77e-05 | 2.81e-05 | 2.81e-04 | 9.50e-06 | 6.52e-04 | 1.32e-05 | 5.45e-03 |
| T = 10 | 2.99e-02 | 1.91e-05 | 2.91e-05 | 3.00e-04 | 1.01e-05 | 7.17e-04 | 1.47e-05 | 5.90e-03 |
| T = 11 | 3.35e-02 | 2.08e-05 | 2.99e-05 | 3.12e-04 | 1.06e-05 | 7.69e-04 | 1.58e-05 | 6.33e-03 |
| T = 12 | 3.71e-02 | 2.19e-05 | 3.07e-05 | 3.25e-04 | 1.09e-05 | 8.21e-04 | 1.65e-05 | 6.67e-03 |
| T = 13 | 4.04e-02 | 2.29e-05 | 3.11e-05 | 3.34e-04 | 1.13e-05 | 8.90e-04 | 1.69e-05 | 6.99e-03 |
| T = 14 | 4.35e-02 | 2.43e-05 | 3.15e-05 | 3.41e-04 | 1.18e-05 | 9.48e-04 | 1.80e-05 | 7.29e-03 |
| T = 15 | 4.67e-02 | 2.53e-05 | 3.22e-05 | 3.47e-04 | 1.22e-05 | 1.02e-03 | 1.87e-05 | 7.53e-03 |
| T = 16 | 4.93e-02 | 2.63e-05 | 3.23e-05 | 3.53e-04 | 1.27e-05 | 1.06e-03 | 1.98e-05 | 7.71e-03 |
| T = 17 | 5.19e-02 | 2.74e-05 | 3.27e-05 | 3.57e-04 | 1.31e-05 | 1.12e-03 | 2.04e-05 | 7.90e-03 |
| T = 18 | 5.40e-02 | 2.88e-05 | 3.28e-05 | 3.60e-04 | 1.35e-05 | 1.17e-03 | 2.07e-05 | 8.03e-03 |
| T = 19 | 5.61e-02 | 2.98e-05 | 3.31e-05 | 3.64e-04 | 1.41e-05 | 1.20e-03 | 2.16e-05 | 8.17e-03 |
| T = 20 | 5.83e-02 | 3.07e-05 | 3.32e-05 | 3.68e-04 | 1.41e-05 | 1.26e-03 | 2.23e-05 | 8.32e-03 |
| T = 21 | 6.04e-02 | 3.15e-05 | 3.35e-05 | 3.72e-04 | 1.46e-05 | 1.30e-03 | 2.27e-05 | 8.44e-03 |
| T = 22 | 6.26e-02 | 3.20e-05 | 3.35e-05 | 3.76e-04 | 1.48e-05 | 1.34e-03 | 2.34e-05 | 8.56e-03 |
| T = 23 | 6.45e-02 | 3.29e-05 | 3.39e-05 | 3.77e-04 | 1.49e-05 | 1.38e-03 | 2.39e-05 | 8.66e-03 |
| T = 24 | 6.62e-02 | 3.36e-05 | 3.41e-05 | 3.79e-04 | 1.52e-05 | 1.42e-03 | 2.41e-05 | 8.78e-03 |
| T = 25 | 6.79e-02 | 3.43e-05 | 3.43e-05 | 3.80e-04 | N/A^a^ | 1.45e-03 | 2.44e-05 | 8.90e-03 |
| T = 26 | 6.92e-02 | 3.50e-05 | 3.45e-05 | 3.83e-04 | N/A^a^ | 1.47e-03 | 2.44e-05 | 8.97e-03 |
| T = 27 | 7.06e-02 | 3.55e-05 | 3.47e-05 | 3.87e-04 | 1.54e-05 | 1.50e-03 | 2.47e-05 | 9.05e-03 |
| T = 28 | 7.24e-02 | 3.63e-05 | 3.49e-05 | 3.89e-04 | 1.56e-05 | 1.52e-03 | 2.49e-05 | 9.11e-03 |
| T = 29 | 7.38e-02 | 3.76e-05 | N/A^a^ | 3.92e-04 | 1.57e-05 | 1.55e-03 | 2.54e-05 | 9.18e-03 |
| T = 30 | 7.49e-02 | 3.78e-05 | 3.50e-05 | 3.93e-04 | 1.57e-05 | 1.56e-03 | 2.59e-05 | 9.24e-03 |

ARF = acute renal failure; CVA = cerebrovascular accident; MI = myocardial infarction; VTE = venous thromboembolism.

^a^The baseline hazard is unavailable for these complications on these specific days due to the absence of outcomes of interests.

$$h\left( t \right)=h_{0}\left( t \right)\times e^\{(b_{1}x_{1}+b_{2}x_{2}+\cdots+b_{p}x_{p})\}$$

*For example, at time t = 30 days, for a patient aged 30 years with a BMI of 28, identified as male, a surgery duration of 170 minutes and all other covariates set at their reference levels, the hazard of readmission is calculated as follows:*

$$h\left( 30 \right)=0.0749*e^{\left\{ \left( - 0.0022 * 30 - 0.0096 - 0.0028 * 28 + 0.0015 * 170 \right) \right\}}=0.083$$

**S2.12 Table.** Comparison of Key Performance Measures Between the Cox Proportional-Hazard Models and Accelerated Failure Time Models.

| **Model** |  | **Harrell’s concordance index** | | **AIC** | | **Log-likelihood** | |
| --- | --- | --- | --- | --- | --- | --- | --- |
|  | ***N*** | **Cox** | **AFT** | **Cox** | **AFT** | **Cox** | **AFT** |
| **Mortality** | 129 606 | 0.8456 | 0.8456 | 14998.1 | 12097.4 | -7464.04 | -6012.69 |
| **Myocardial infarction** | 129 677 | 0.8128 | 0.8128 | 14081.0 | 11186.0 | -7005.48 | -5555.99 |
| **Cerebrovascular event** | 126 943 | 0.8021 | 0.8021 | 4849.51 | 4426.16 | -2392.75 | -2180.08 |
| **Acute renal failure** | 129 606 | 0.8062 | 0.8061 | 7722.30 | 6732.18 | -3826.15 | -3330.09 |
| **Pneumonia** | 129 677 | 0.7726 | 0.7726 | 31656.7 | 23450.6 | -15793.3 | -11688.3 |
| **Sepsis** | 129 676 | 0.6790 | 0.6789 | 76891.7 | 52174.9 | -38410.9 | -26050.5 |
| **Readmission** | 129 643 | 0.6173 | 0.6173 | 260643 | 150860 | -130287 | -75392.9 |
| **Venous thromboembolism** | 129 676 | 0.6546 | 0.6547 | 34811.4 | 26205.5 | -17370.7 | -13065.8 |

AFT, accelerated failure time model; AIC, Akaike Information Criterion; Cox, Cox proportional-hazard model; *N* = total number of patients.
