# Supplementary material for: Development and internal validation of time-to-event risk prediction models for major medical complications within 30 days after elective colectomy
Source: PLoS One. 2024 Dec 2;19(12):e0314526. doi: 10.1371/journal.pone.0314526 (PMC11611139; doi:10.1371/journal.pone.0314526)
Supplement: S3 Appendix — (DOCX) [file pone.0314526.s003.docx]

**Appendix 3. Supplemental Figures**

**S1 Fig.** Cumulative Hazard Plots for Morbidity and Mortality Outcomes.

**
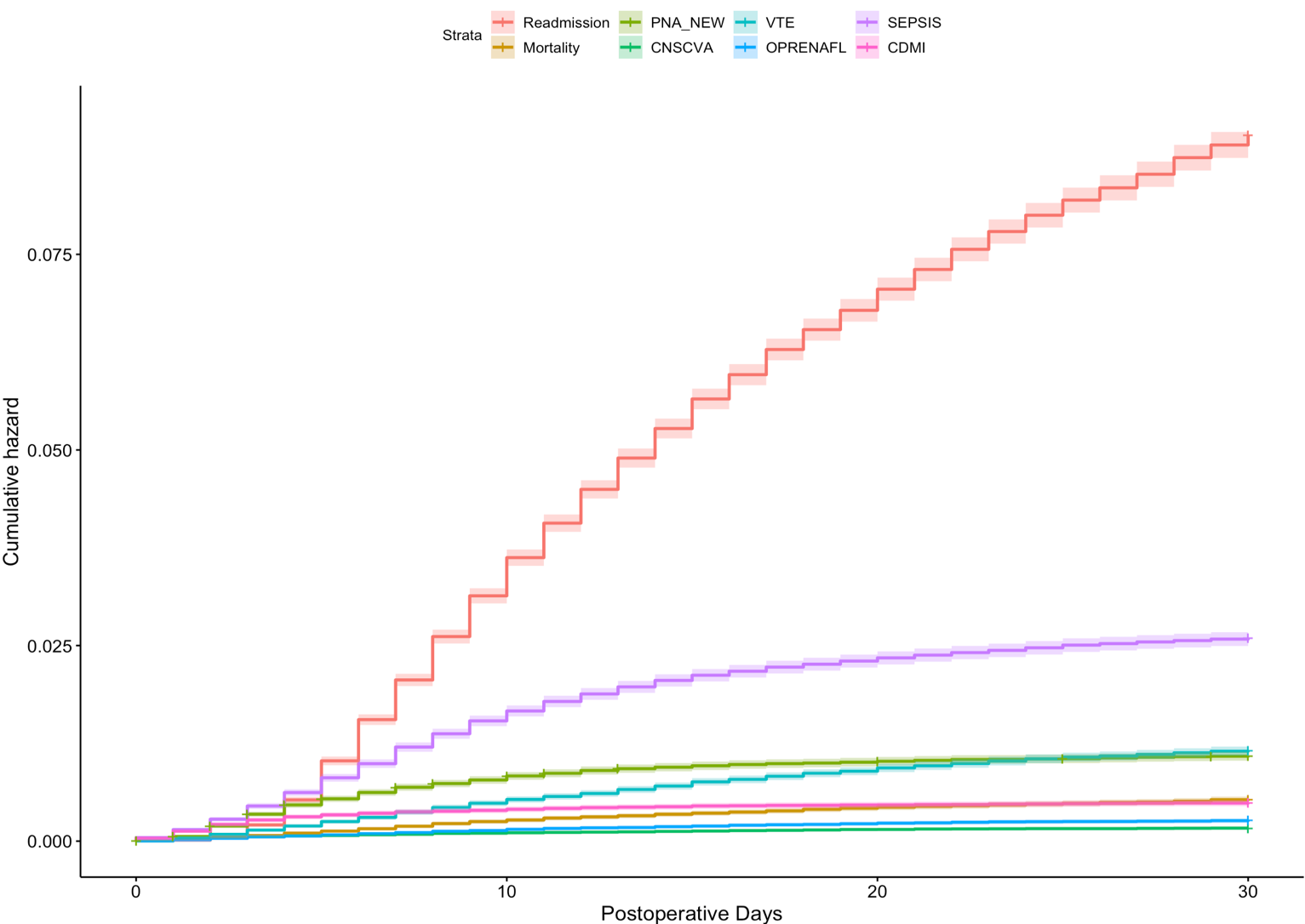
**

**S2 Fig.** Calibration Plots for the Cox Proportional Hazards Models for Outcomes.

1. **Mortality

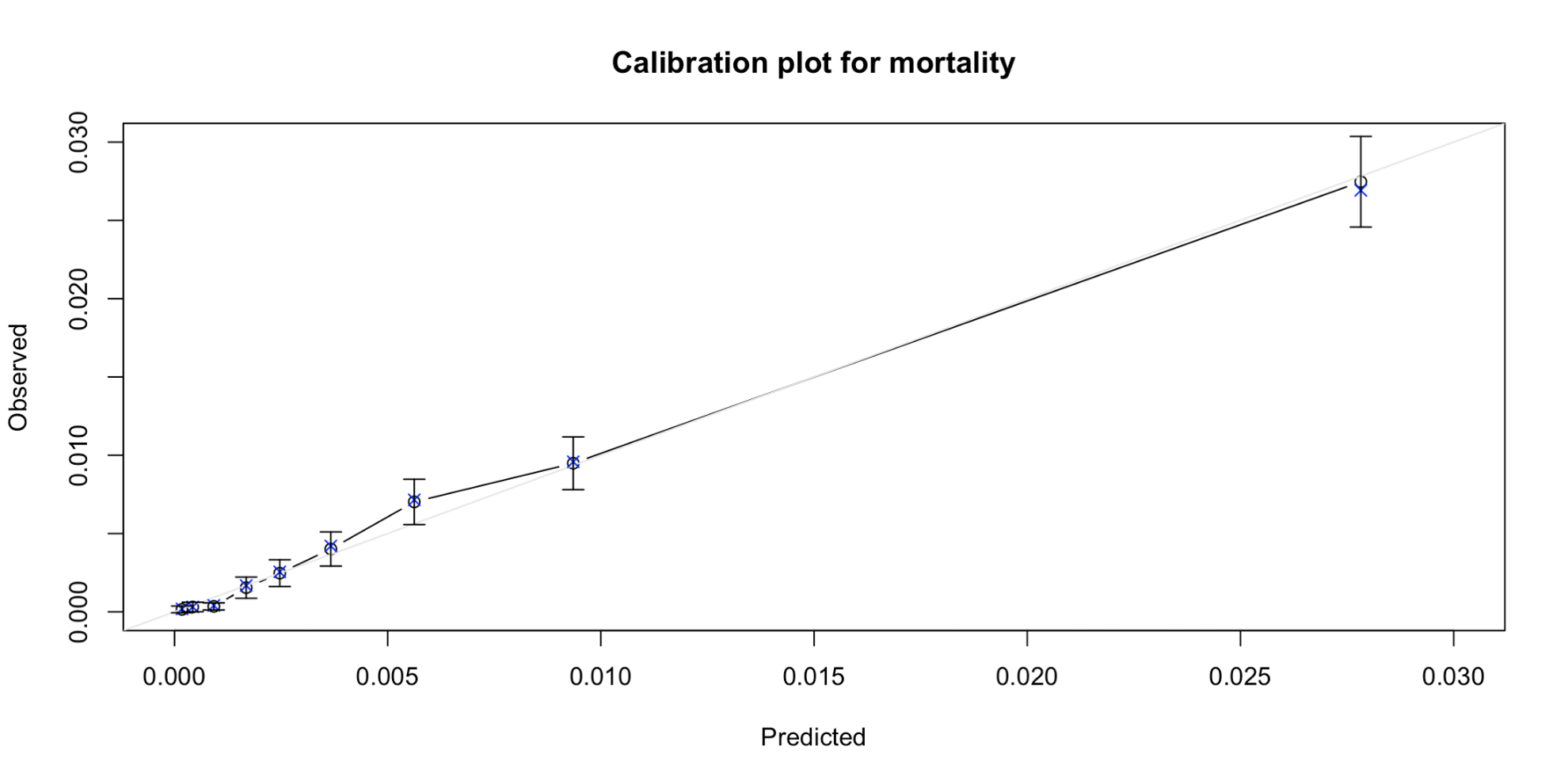
**
2. **Myocardial infarction

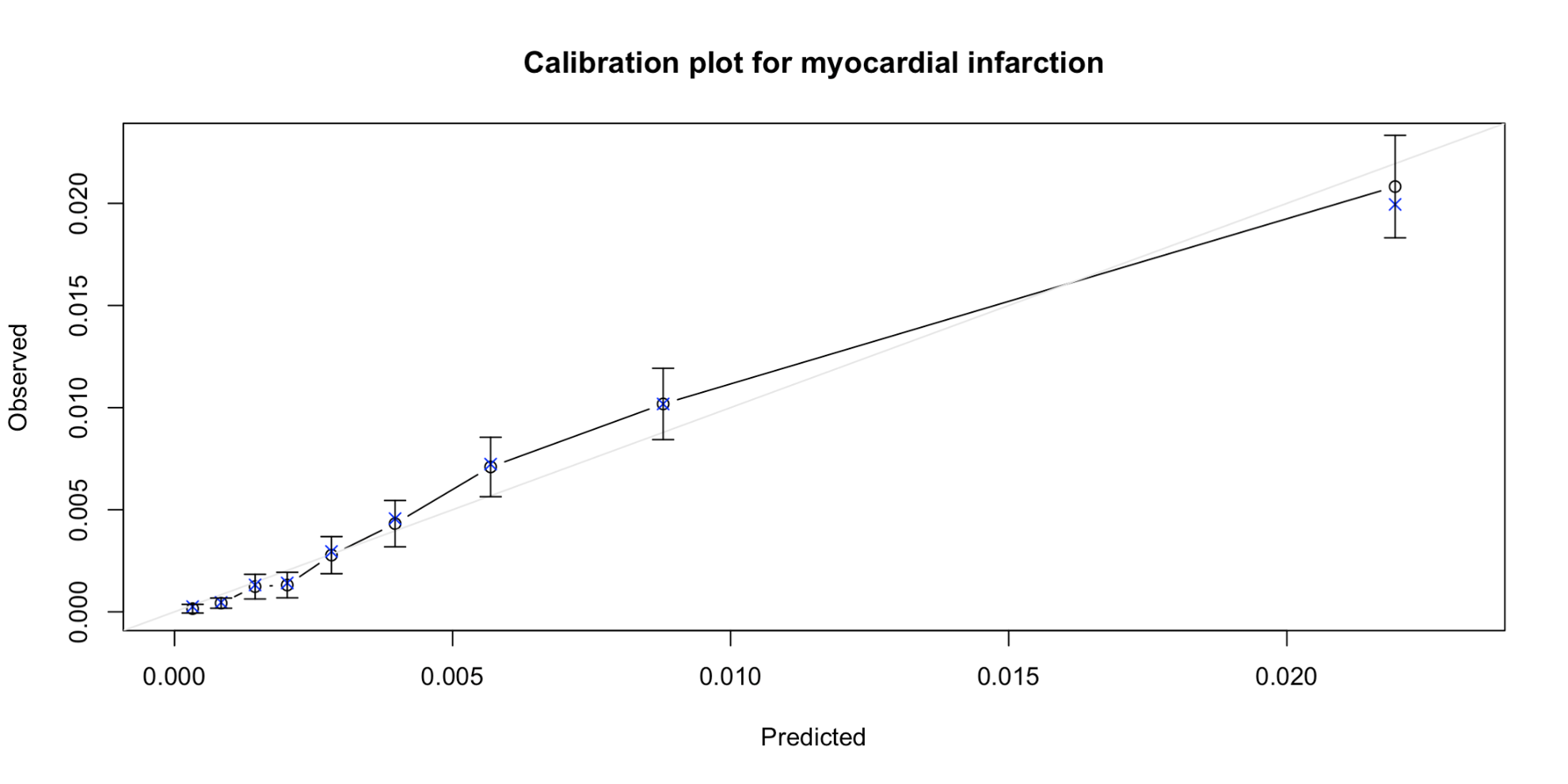
**
3. **Cerebrovascular accident**

**
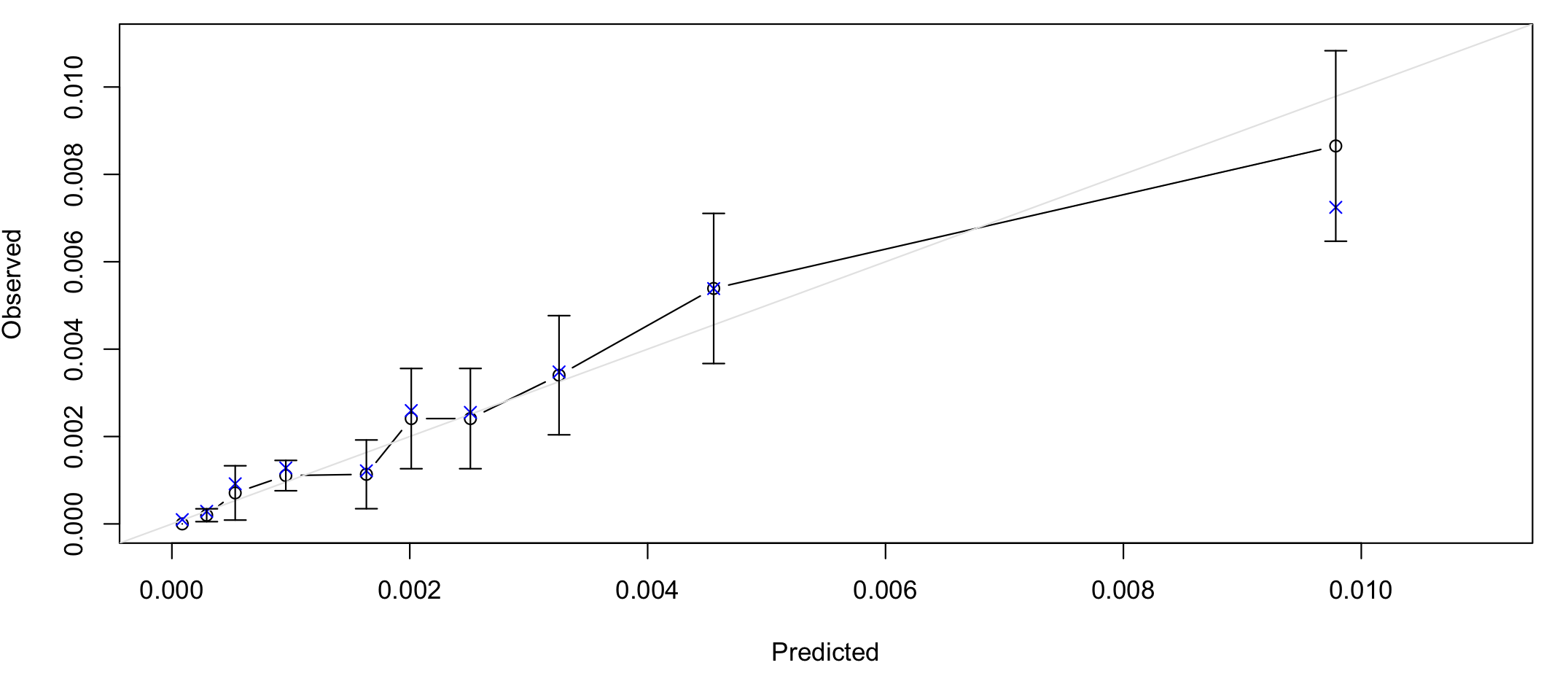
**

1. **Acute renal failure

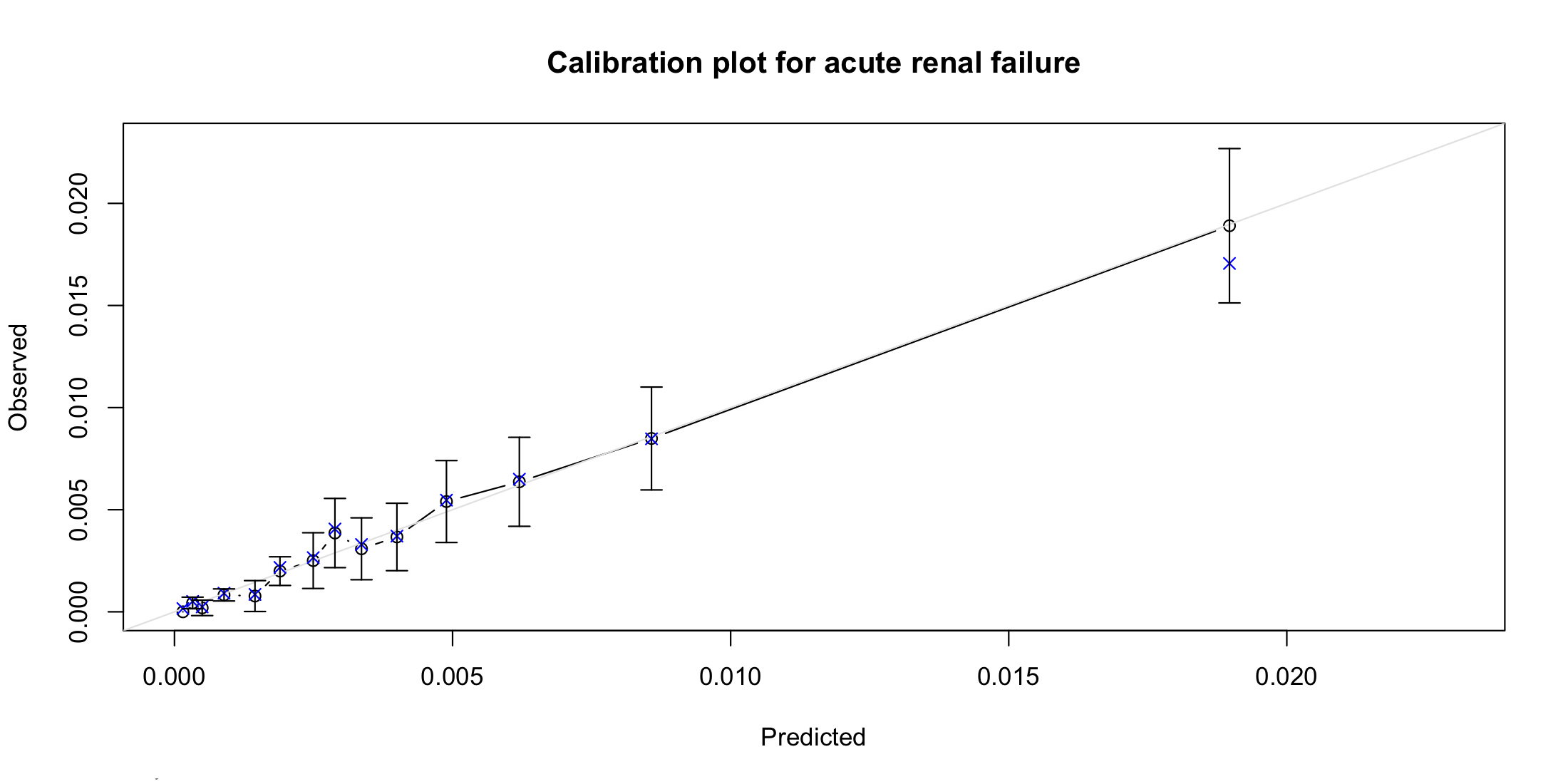
**
2. **Pneumonia

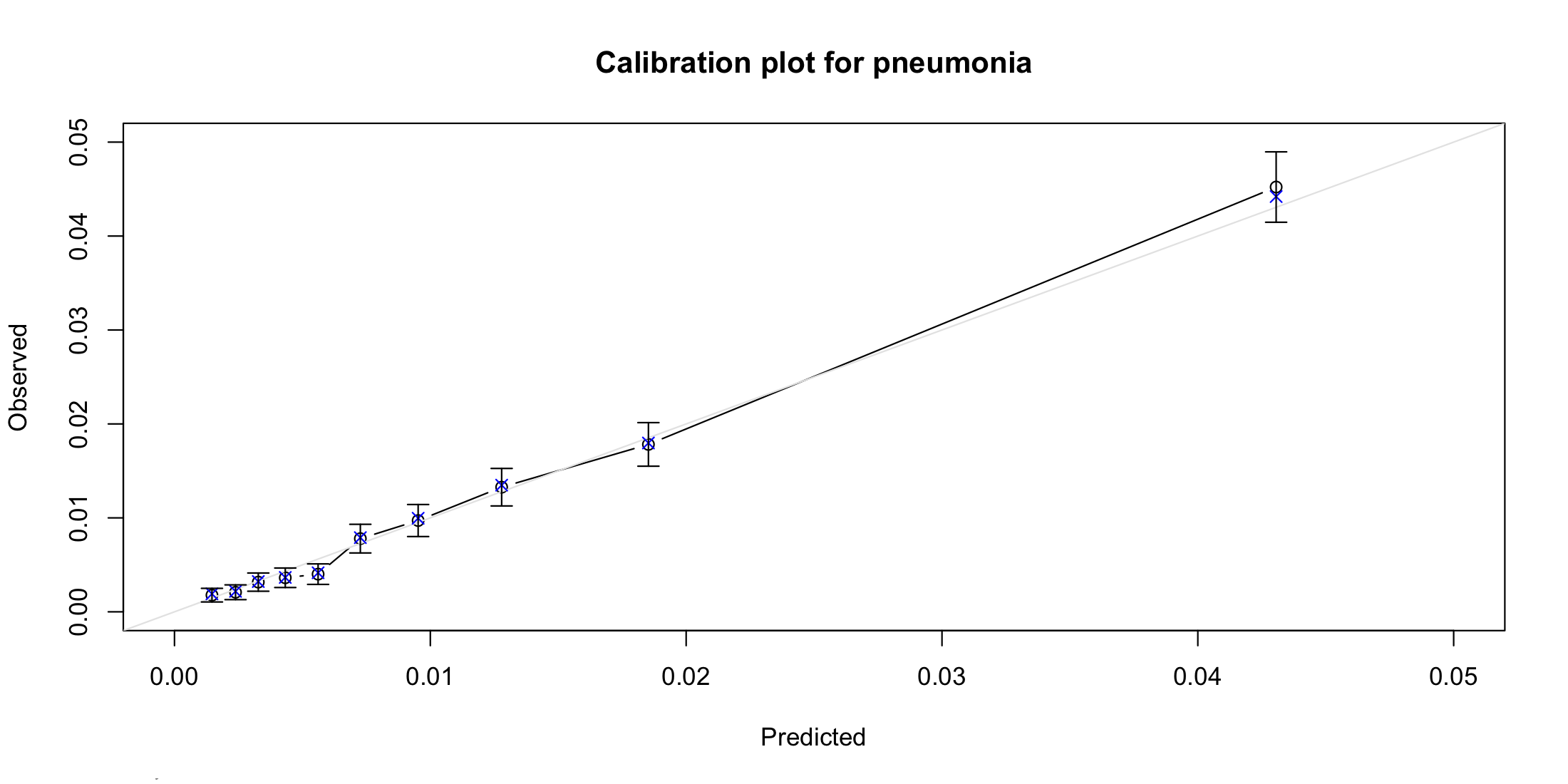
**
3. **Sepsis

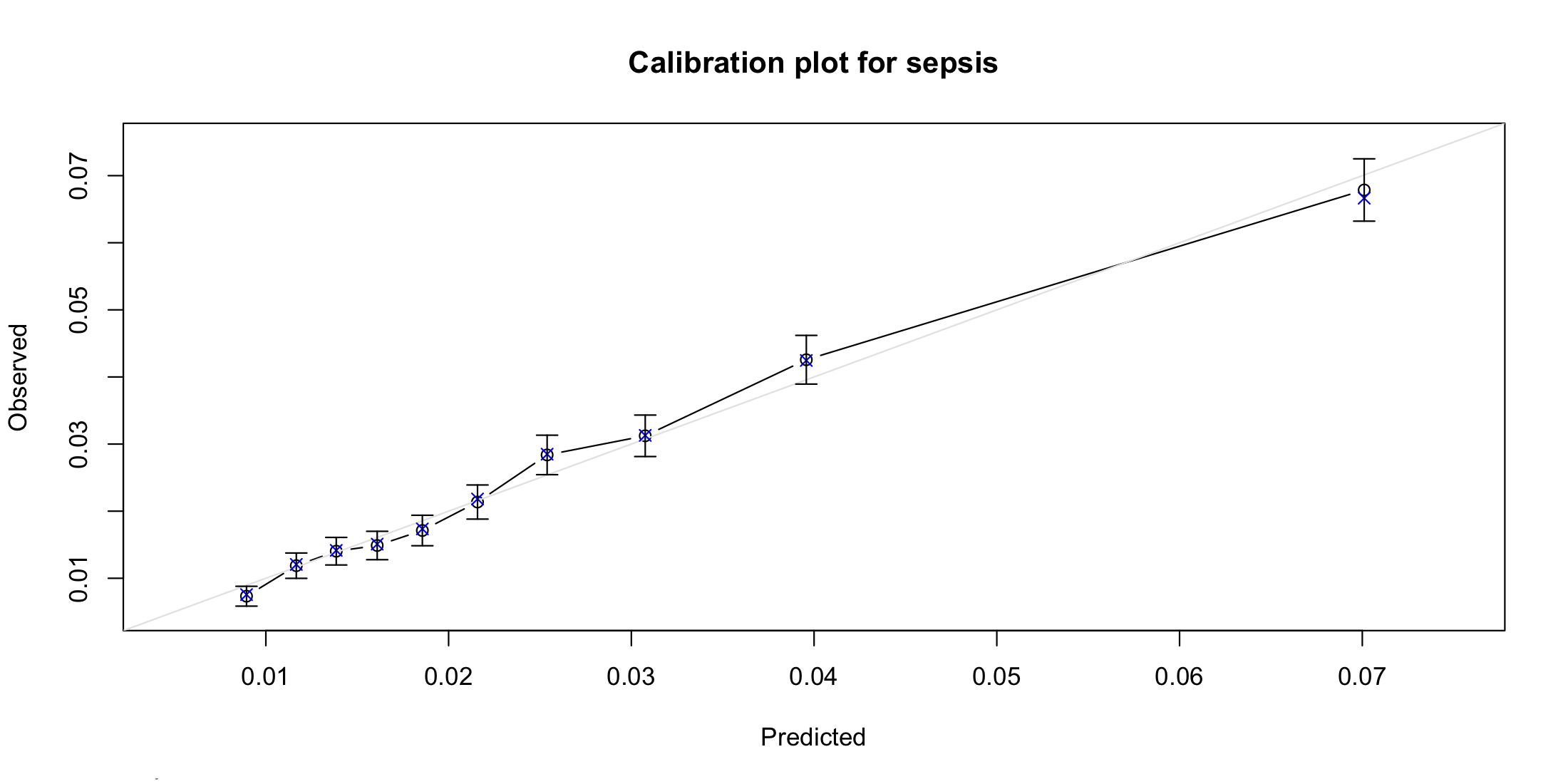
**
4. **Readmission

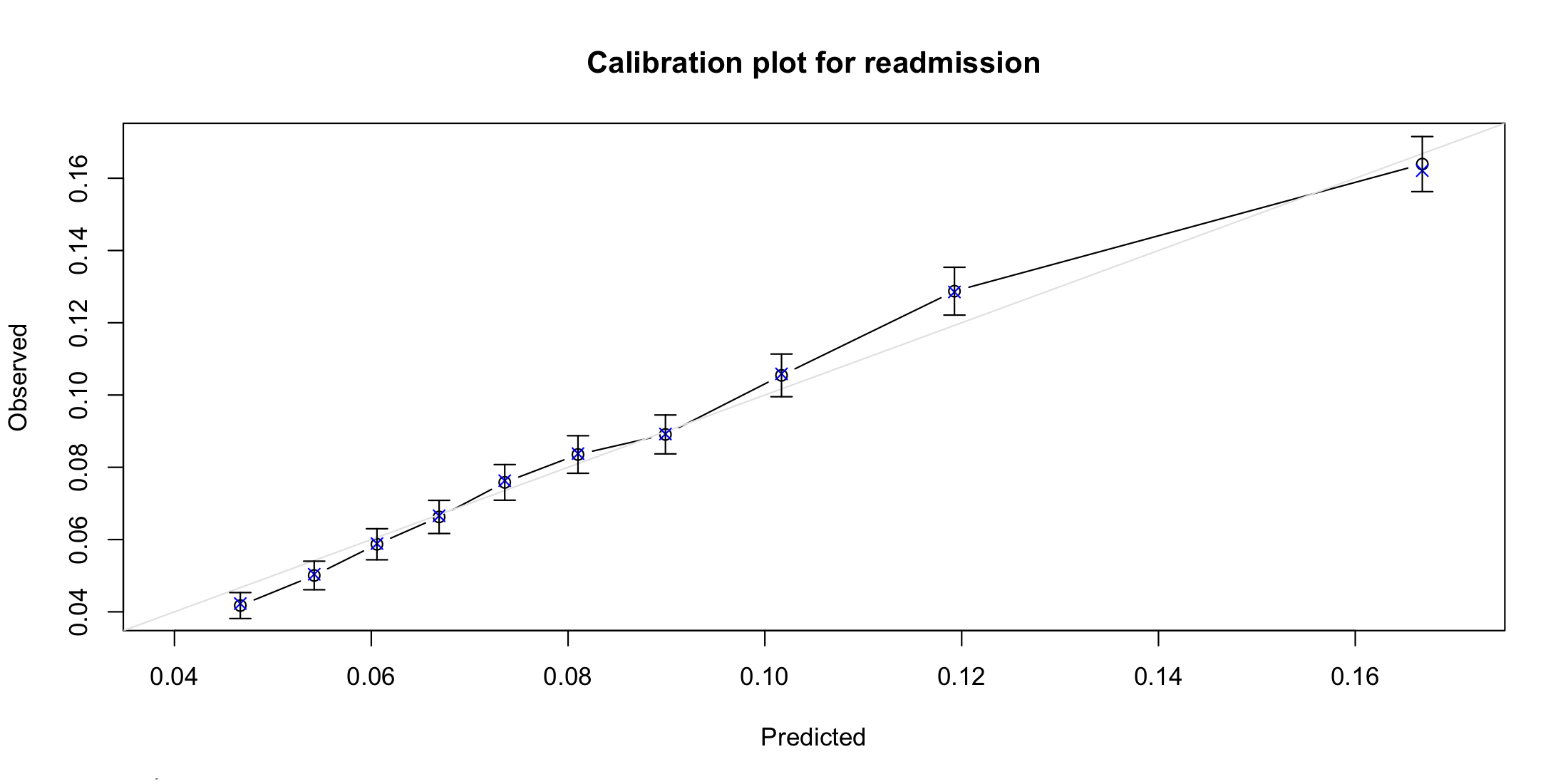
**
5. **Venous thromboembolism

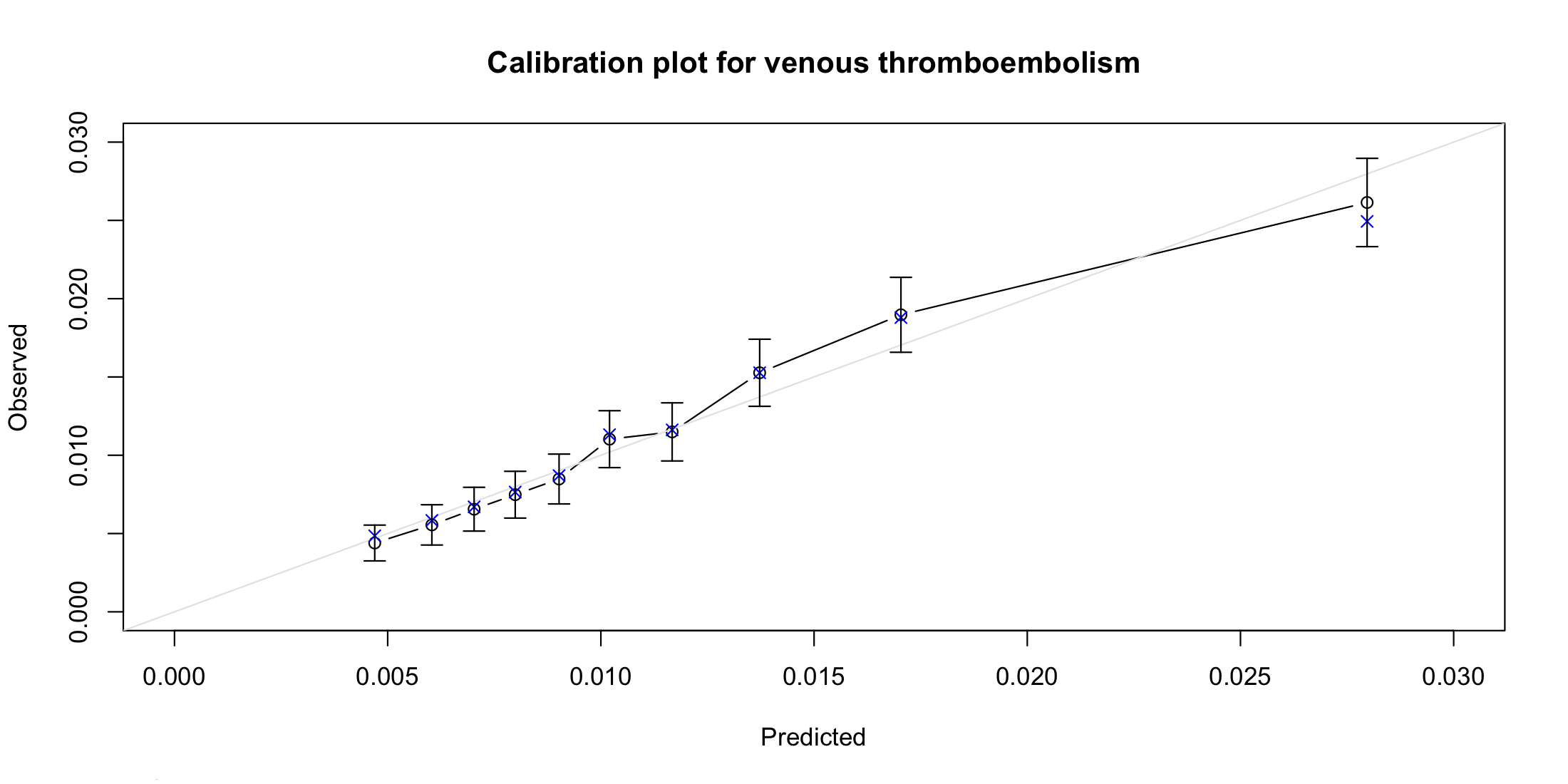
**
