# Supplementary material for: Development and internal validation of time-to-event risk prediction models for major medical complications within 30 days after elective colectomy
Source: PLoS One. 2024 Dec 2;19(12):e0314526. doi: 10.1371/journal.pone.0314526 (PMC11611139; doi:10.1371/journal.pone.0314526)
Supplement: S4 Appendix — (DOCX) [file pone.0314526.s004.docx]

**Appendix 4. Example patient illustration of predictions.** The attached example is based on a patient with ASA classification II, primary indication for surgery as cancer, and wound classification as clean/contaminated, with all other factors set to reference level.

**
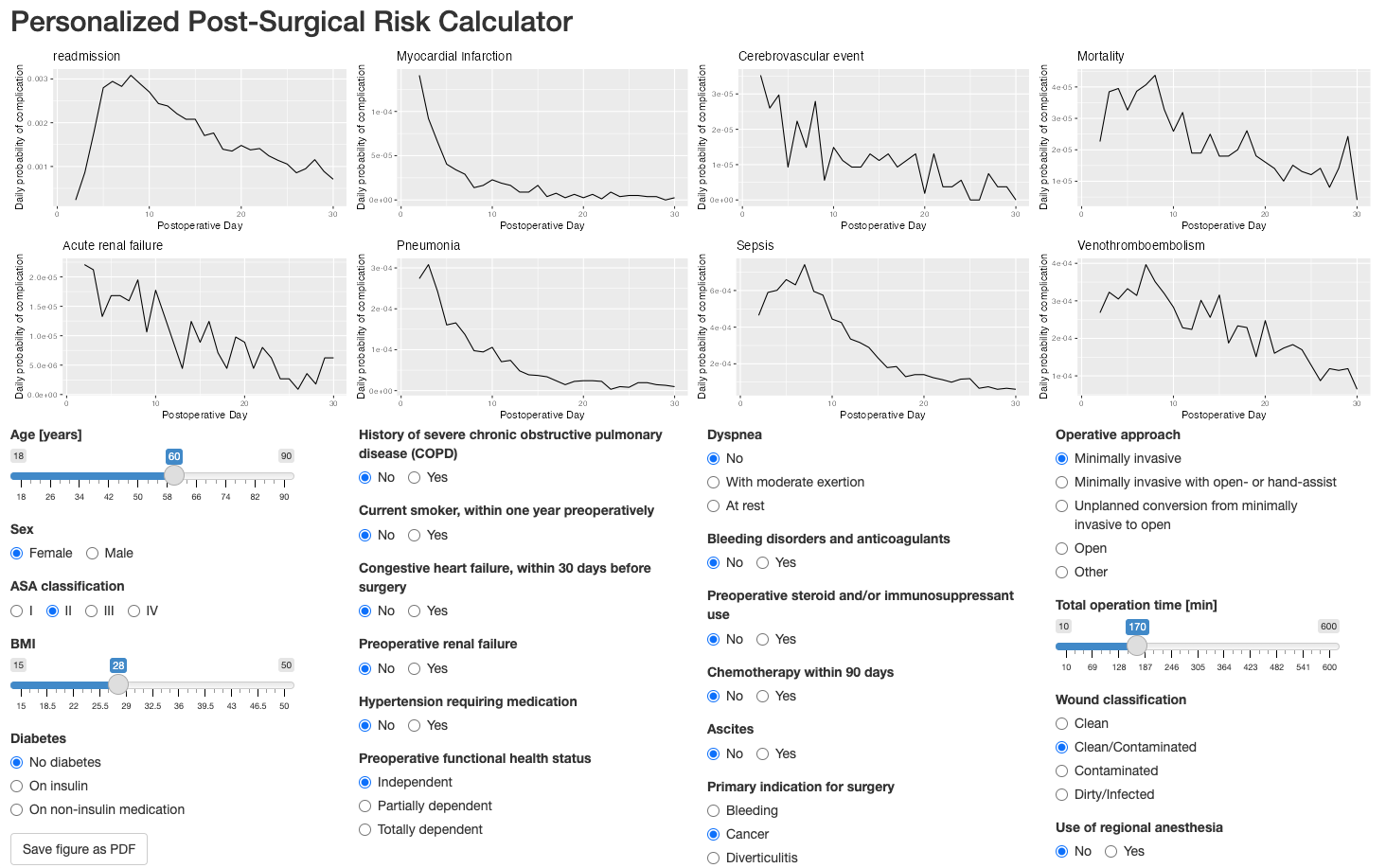
**
