# Supplementary material for: Development and internal validation of time-to-event risk prediction models for major medical complications within 30 days after elective colectomy
Source: PLoS One. 2024 Dec 2;19(12):e0314526. doi: 10.1371/journal.pone.0314526 (PMC11611139; doi:10.1371/journal.pone.0314526)
Supplement: S5 Appendix — (DOCX) [file pone.0314526.s005.docx]

**Appendix 5.** Sensitivity analysis for sepsis and readmission models

| **Model** | **AIC** | **Harrell’s concordance index (SE)^a^** | **Uno’s concordance index** | **Time-dependent AUROC at 30 days (SE)^a^** | **Cox and Snell’s** **Pseudo R-squared** | **Maximum Possible  Pseudo R-squared** **^b^** | **Scaled time-dependent Brier score at 30 days** |
| --- | --- | --- | --- | --- | --- | --- | --- |
| **Sepsis** | 76891.72 | 0.679 (0.0046) | 0.679 | 0.670 (0.0044) | 0.0111 | 0.4532 | 0.0142 |
| **Sepsis (AIC stepwise)** | 76880.91 | 0.679 (0.0043) | 0.679 | 0.669 (0.0042) | 0.0111 | 0.4532 | 0.0142 |
| **Readmission** | 260643.4 | 0.617 (0.0028) | 0.617 | 0.789 (0.0077) | 0.0149 | 0.8680 | 0.0165 |
| **Readmission (AIC stepwise)** | 260638.8 | 0.617 (0.0025) | 0.617 | 0.789 (0.0072) | 0.0149 | 0.8680 | 0.0165 |

Comparison of key performance measures between the Cox Proportional-Hazard models with and without stepwise selection by Akaike Information Criterion for sepsis and readmission. For the sepsis stepwise model, the predictors eliminated were age, body mass index, diabetes, congestive heart failure, chemotherapy within 90 days preoperatively, and use of any regional anesthesia. For the readmission model, the predictors eliminated were sex, dyspnea, and use of any regional anesthesia~~.~~

AIC, Akaike Information Criterion; Cox, Cox proportional-hazard model; *N* = total number of patients.
